# Mechanochemical synthesis of *N*-salicylidene­aniline: thermosalient effect of polymorphic crystals

**DOI:** 10.1107/S2052252517004043

**Published:** 2017-03-27

**Authors:** Sudhir Mittapalli, D. Sravanakumar Perumalla, Ashwini Nangia

**Affiliations:** a School of Chemistry, University of Hyderabad, Prof. C. R. Rao Road, Gachibowli, Hyderabad 500 046, India; b Department of Inorganic and Physical Chemistry, Indian Institute of Science, Bengaluru, India; c CSIR-National Chemical Laboratory, Dr Homi Bhabha Road, Pune 411 008, India

**Keywords:** polymorphism, halogen bonding, materials science, crystal engineering, intermolecular interactions, mechanochemistry, crystal design, hydrogen bonding

## Abstract

Polymorphs of the dichloro derivative of *N*-salicylideneaniline exhibit mechanical responses such as jumping (Forms I and III) and exploding (Form II) in its three polymorphs. The molecules are connected *via* the amide N—H⋯O dimer synthon and C—Cl⋯O halogen bond in the three crystal structures. A fourth high-temperature Form IV was confirmed by variable-temperature single-crystal X-ray diffraction at 180°C. The behaviour of jumping exhibited by the polymorphic crystals of Forms I and III is due to the layered sheet morphology and the transmission of thermal stress in a single direction, compared with the corrugated sheet structure of Form II such that heat dissipation is more isotropic causing blasting. The role of weak C—Cl⋯O interactions in the thermal response of molecular crystals is discussed.

## Abstract 2

Among the halogen derivatives of *N*-salicylideneaniline, polymorphs of the dichloro compound *A* showed the mechanical response of jumping (Forms I and III) and sudden blasting (Form II) upon heating. This difference is ascribed to the layered packing in Forms I and III such that the thermal stress acts in a unidirectional manner while for the corrugated layer structure of Form II the outcome is blasting because the thermal energy dissipates in different directions.

## 1. Introduction   

The construction of mechanically responsive materials which exhibit durability, reversibility and good stability is a challenging task (Ramirez *et al.*, 2013 ▸; Panda *et al.*, 2014 ▸). Structural studies on mechanically responsive materials have gained importance due to their wide ranging applications in materials science and medicine (Liu *et al.*, 2014 ▸). Solids which are responsive to external stimuli such as heat, pressure, humidity, light and can convert them efficiently into work are potential candidates for development as smart materials, *e.g.* artificial muscles, actuators, biomimetic and technomematic materials (Liu *et al.*, 2014 ▸; Lehn, 2002 ▸; Ikeda *et al.*, 2007 ▸; Sagara & Kato, 2009 ▸; Rowan, 2009 ▸; Takashima *et al.*, 2012 ▸; Mather, 2007 ▸; Sato, 2016 ▸).The efficient conversion of light and heat energy into mechanical work by dense and orderly packed crystals was reviewed recently (Naumov *et al.*, 2015 ▸). Mechanical response occurs when the material is heated, or exposed to light/pressure, that causes an increase in the strain inside the crystal lattice. Thermosalient effects of (phenyl­azophenyl)-Pd-(hexafluoroacetonate) complex was first reported by Etter & Siedle (1983 ▸). Recently, Panda *et al.* (2014 ▸) revisited the same complex and observed positive and negative thermal expansion and thermosalient effects. Desiraju and co-workers (Ghosh *et al.*, 2015 ▸) reported that thermosalient effects are mainly due to a sharp phase transition and anisotropy in structural parameters. Vittal’s group reported photo actuation in Zn metal complexes due to an increase in strain by sudden expansion during 2 + 2 photo cycloaddition reaction (Medishetty *et al.*, 2014 ▸). Naumov’s group (Sahoo *et al.*, 2013 ▸; Nath *et al.*, 2014 ▸, 2015 ▸) analyzed thermosalient crystals and classified them into three classes. If the molecule does not have any strong hydrogen-bonding donor and acceptor groups, then it is classified as Class I. Class II consists of molecules with hydrogen-bonding groups in a crowded environment, which will not allow strong hydrogen bonding, and Class III molecules have good hydrogen-bonding functional groups which participate in strong hydrogen bonds. There are a few examples of both thermomechanical and photomechanical effects in crystals (Naumov *et al.*, 2015 ▸; Lusi & Bernstein, 2013 ▸; Skoko *et al.*, 2010 ▸; Medishetty *et al.*, 2015 ▸; Mishra *et al.*, 2015 ▸; Takeda & Akutagawa, 2016 ▸; Lieberman *et al.*, 2000 ▸; Brandel *et al.*, 2015 ▸).

Schiff’s bases and their metal complexes find wide applications in biological systems, as well as non-linear optical materials, in electrochemical cells, as corrosion inhibitors, thermo-/photochromic materials and as thermally/chemically resistant flame retardants (Jia & Li, 2015 ▸; Singh *et al.*, 2014 ▸). In the present investigation, we synthesized dichloro (compound A), dibromo (compound B) and diiodo (compound C) derivatives of *N*-salicylideneaniline (Schiff’s bases) (Scheme 1). The chemical structures (Fig. S1 of the supporting information) and procedures followed for the synthesis of compounds are given in §2. The presence of a strong hydrogen bonding functional group (*e.g.* the amide group) makes Compound A a Class III category, and the surprising effects of jumping, breaking and sudden blasting upon heating its polymorphic crystals are presented in this paper. To our knowledge, such a phenomenon is unusual and not reported in polymorphs of the same compound (Steiner *et al.*, 1993 ▸; Crawford *et al.*, 2007 ▸; Sahoo *et al.*, 2013 ▸).
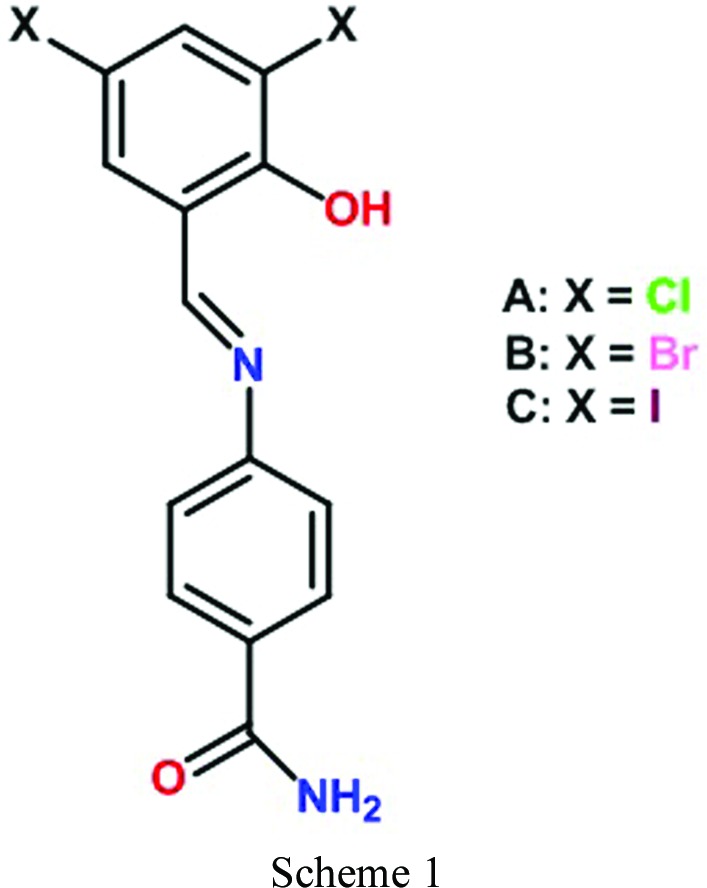


## 2. Experimental   

For the synthesis of compound A ((*E*)-4-((3,5-dichloro-2-hydroxybenzylidene)amino)benzamide) two methods were followed: the first is mechanochemical (Zbačnik & Kaitner, 2014 ▸) grinding and the second is a conventional reaction with azeotropic removal of water (Scheme S1) (Safin *et al.*, 2014 ▸).

Method 1: The product was obtained by mechanical grinding of 4-aminobenzamide and 3,5-dichloro­salicylaldehyde in a 1:1 stoichiometric ratio (1 mmol each, 136 mg; 191 mg) by adding 2–3 ml of methanol (drop by drop addition) solvent for 30 min and the resulting product was washed with hexane 3–4 times to remove unwanted by-products. The product salicylideneaniline was finally purified by crystallization from methanol and characterized by ^1^H-NMR and ^13^C-NMR, FT–IR (Figs. S24–26) and finally confirmed by single-crystal X-ray diffraction. We followed the same procedure for the synthesis of compound B and compound C and confirmed their structures by ^1^H-NMR and single-crystal XRD.

Method 2: 4-Aminobenzamide 1 mmol (136.15 mg) was dissolved in anisole (10 ml) in a 100 ml round-bottom flask fitted with a Dean-Stark setup. 1 mmol of 3,5-dichloro­salicylaldehyde (191.01 mg) was added to the solution. The reaction mixture turns to a light yellow color (after 10 min) and it was refluxed for 4 h. The solution was concentrated by removing the solvent and then washed with *n*-hexane 3–4 times to remove unwanted by-products. Red color crystals were obtained in 80% yield. We followed the same procedure for the synthesis of compound B and compound C and confirmed their structures by ^1^H-NMR and single-crystal X-ray diffraction.

## 3. Compound A   

^1^H-NMR (400 MHz, DMSO-*d*_6_, 298 K, δ in p.p.m.; Fig. S24): 14.22 (s, 1H), 9.08 (s, 1H), 8.06 (br, 1H), 8.01 (d, *J* = 8.4 Hz, 2H), 7.78 (dd, *J* = 10.4 Hz and *J* = 2.4 Hz, 2H), 7.56 (d, *J* = 8.4 Hz, 2H), 7.45 (br, 1H).

^13^C-NMR (400 MHz, DMSO-*d*_6_, 298 K, δ in p.p.m.; Fig. S25): 167.5, 163.8, 156.3, 149.1, 133, 133, 131.3, 131.3, 129.42, 122.6, 121.8 and 120.9.

FT–IR (KBr, cm^−1^; Fig. S26): 3493.3 (s, N—H), 3164 (m, br, OH), 1594.7 (s, C=O), 1562.8 (m, C=N), 1453.6 (w), 1415.4 (w), 1393.4 (m), 1262 (w), 1198 (w), 1176 (m).

Thermogravimetric analysis (TGA) showed no weight loss or decomposition on heating of the material (Fig. S27).

## 4. Compound B   

^1^H-NMR (400 MHz, DMSO-*d*_6_, 298 K, δ in p.p.m., Fig. S28): 14.38 (s, 1H), 9.05 (s, 1H), 8.03 (d, 2H), 7.98 (d, *J* = 4.2 Hz, 2H), 7.94 (d, *J* = 2.8 Hz, 2H), 7.55 (d, *J* = 4.2 Hz, 2H), 7.42 (br, 1H).

## 5. Compound C   

^1^H-NMR (400 MHz, DMSO-*d*_6_, 298 K, δ in p.p.m., Fig. S29): 14.50 (s, 1H), 8.96 (s, 1H), 8.17 (s, 1H), 8.028 (d, *J* = 8.0 Hz, 2H), 7.99 (d, *J* = 4.2 Hz, 2H), 7.56 (d, *J* = 4.4 Hz, 2H), 7.43 (br, 1H).

### 5.1. X-ray crystallography   

X-ray reflections for compound A (Form I, Form II and Form III) and compound B were collected on an Oxford Xcalibur Gemini Eos CCD diffractometer at 298 K using Cu *K*α radiation (λ = 1.54184 Å) and data reduction was performed using *CrysAlisPro* (*CrysAlis CCD* and *CrysAlis RED*; Oxford Diffraction, 2008 ▸) and *OLEX*2 (Dolomanov *et al.*, 2009 ▸) to solve and refine the crystal structure. X-ray reflections for compound C were collected on a Bruker D8 Quest CCD diffractometer equipped with a graphite monochromator and Mo *K*α fine-focus sealed tube (λ = 0.71073 Å), and high-temperature Form IV (Compound A) was collected on Bruker D8 venture diffractometer at 180°C; reduction was performed using *APEX*II Software (Bruker, 2000 ▸). Intensities were corrected for absorption using *SADABS* (Sheldrick, 1997 ▸) and the structure was solved and refined using *SHELX*97 (Sheldrick, 1997 ▸). All non-hydrogen atoms were refined anisotropically. Hydrogen atoms on hetero atoms were located from difference electron-density maps and all C—H H atoms were fixed geometrically. Hydrogen-bond geometries were determined in *PLATON* (Spek, 2002 ▸). *X-Seed* (Barbour, 1999 ▸) was used to prepare packing diagrams. Crystal structures are deposited as part of the supporting information and may be accessed at https://www.ccdc.cam.ac.uk/structures/ (CCDC Nos. 1488992–1488996).

### 5.2. Powder X-ray diffraction   

Powder X-ray diffraction was recorded on Bruker D8 Advance diffractometer (Bruker-AXS, Karlsruhe, Germany) using Cu *K*α X-radiation (λ = 1.5406 Å) at 40 kV and 30 mA power. X-ray diffraction patterns were collected over the 2θ range 5–40° at a scan rate of 1° min^−1^.

### 5.3. Vibrational spectroscopy   

A Nicolet 6700 FT–IR spectrometer with a NXR FT–Raman Module was used to record IR spectra. IR spectra were recorded on samples dispersed in KBr pellets.

### 5.4. Thermal analysis   

Differential scanning calorimetry (DSC) was performed on a Mettler Toledo DSC 822e module. Samples were placed in crimped but vented aluminium sample pans. The typical sample size is 3–5 mg; the temperature range was 30–300°C at 20°C min^−1^. Samples were purged by a stream of nitrogen flowing at 60 ml min^−1^.

TGA was carried out using Mettler Toledo TGASDTA851e operating with *STAR* ^e^ software to detect solvates and thermal degradation. Accurately weighed (5–15 mg) samples were loaded in alumina crucibles and heated at a rate of 20°C min^−1^ over a temperature range of 30 to 300°C under a nitrogen purge of 60 ml min^−1^.

## 6. Result and discussion   

### 6.1. X-ray crystal structures   

**Figure 1:**
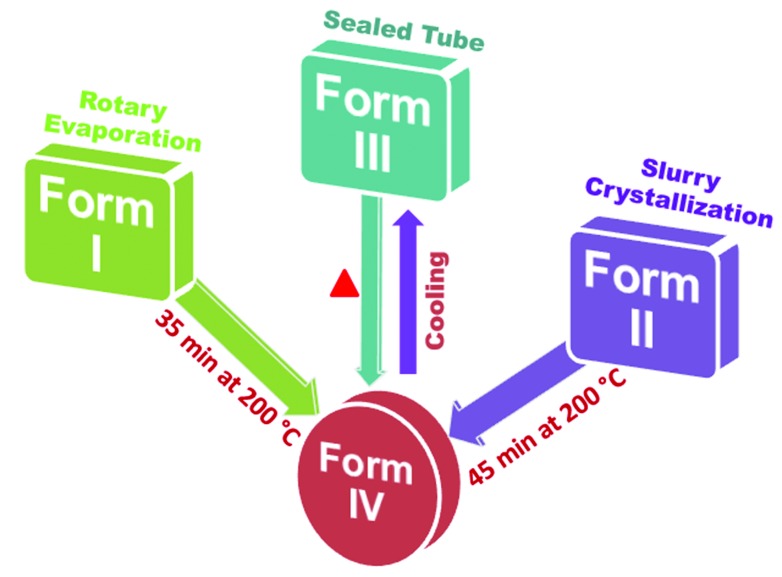
Schematic representation of the preparation conditions and transformations for polymorphs of compound A.

**Figure 2:**
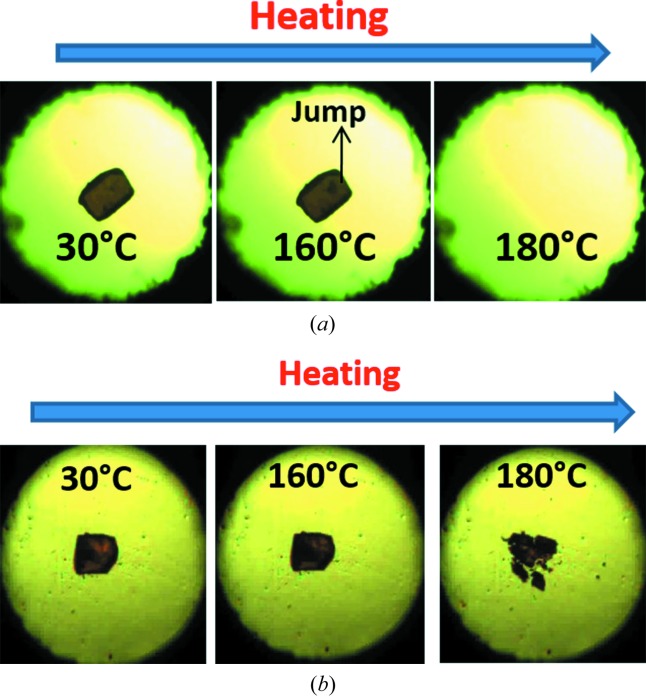
(*a*) Heating of Form I crystal of compound A, and the sudden disappearance of crystal from the hot stage. (*b*) Blasting of Form II crystals on heating.

Compound A was purified by crystallization from methanol, and after multiple recrystallizations diffraction quality red color crystals were harvested from the same solvent. Further screening in different solvents afforded three polymorphs concomitantly from methanol solvent as well as a fourth high-temperature polymorph. Studies on compounds B and C are ongoing, but with similar efforts at crystallization, we have so far obtained a single-crystal structure only for the bromo and iodo salicylideneanilines (no polymorphism). The subsequent discussion is on the structures and properties of chloro compound A. Polymorphs of A in bulk quantity (several mg up to g) were obtained by different techniques, such as Form I by rotary evaporation, Form II from slurry in methanol, and Form III from acetone at 80°C in a sealed tube (Fig. 1 ▸). The preliminary observations in this study were done on a hot stage microscope (HSM). Upon heating Form I crystals to about 170–180°C, a few crystals were seen to suddenly fly off from the stage (about 2–3 cm height) and they moved outside of the camera zone (Fig. 2 ▸ *a*). Form II crystals showed sudden blasting (mini explosion, Fig. 2 ▸ *b*) at 180°C, and Form III crystals behaved similar to those of Form I in that they were flying off suddenly from the hot stage at 180–190°C.

**Figure 3:**
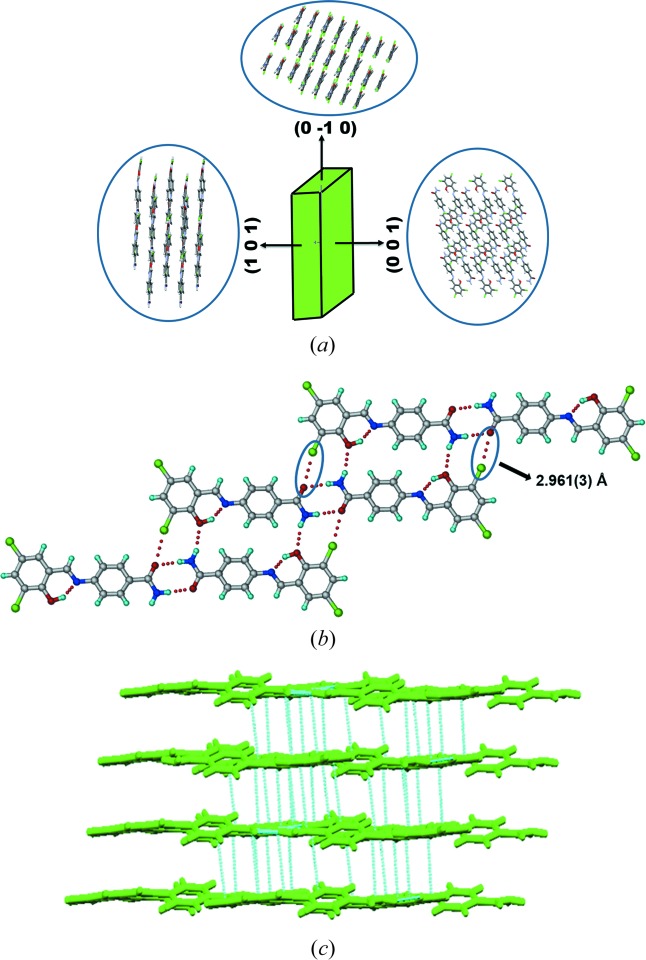
(*a*) Crystal morphology of Compound A Form I and arrangement of molecules in different planes. (*b*) Amide dimers extend through C—Cl⋯O and N—H⋯O interactions (Form I). (*c*) Figure showing the layered arrangement of molecules in Form I crystal structure.

**Figure 4:**
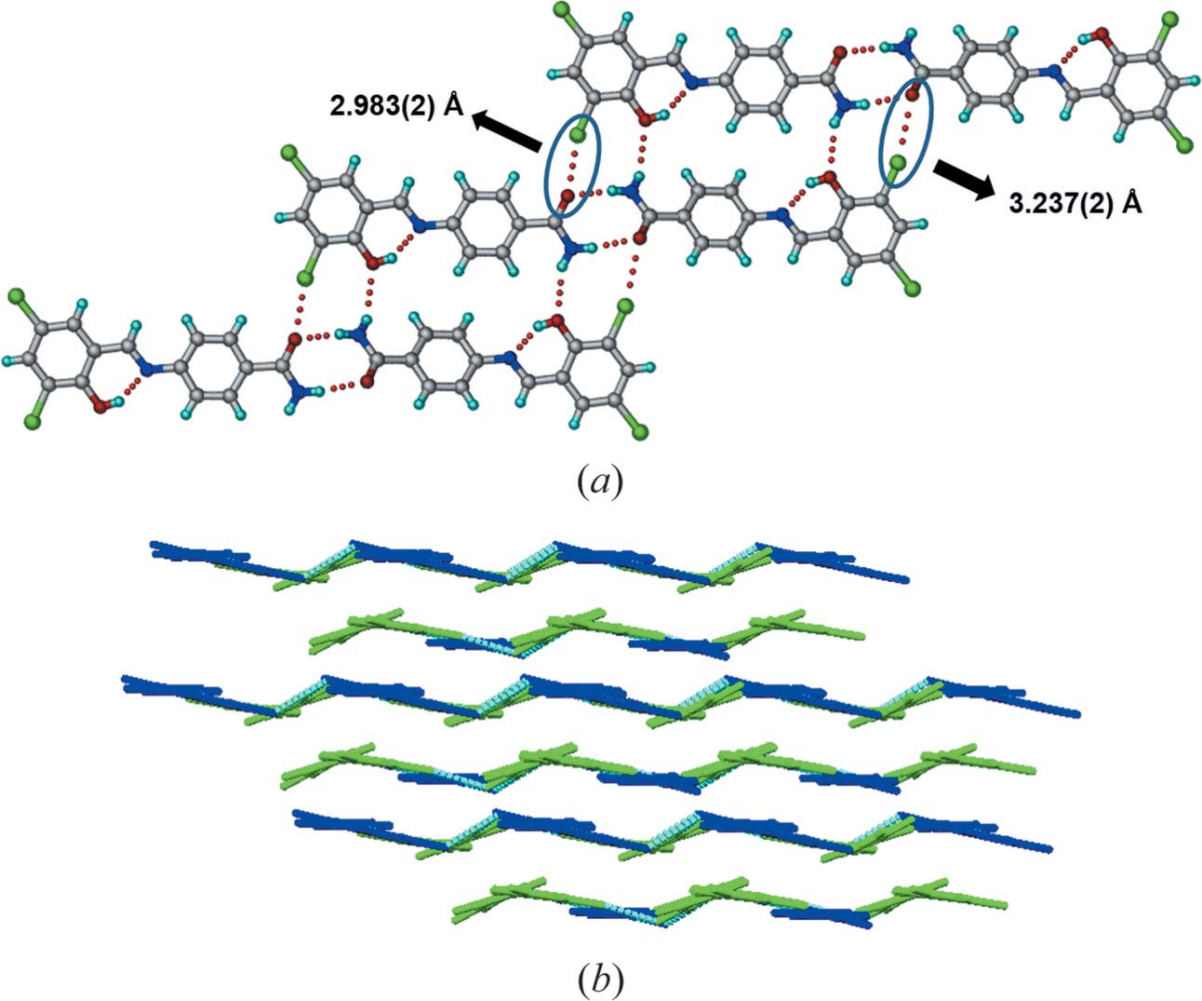
(*a*) Figure showing amide–amide dimers extended by C—Cl⋯O and N—H⋯O interactions in Form II structure. (*b*) Symmetry-independent molecules (indicated in blue and green colors) were arranged in a corrugated wave-like manner in Form II.

**Figure 5:**
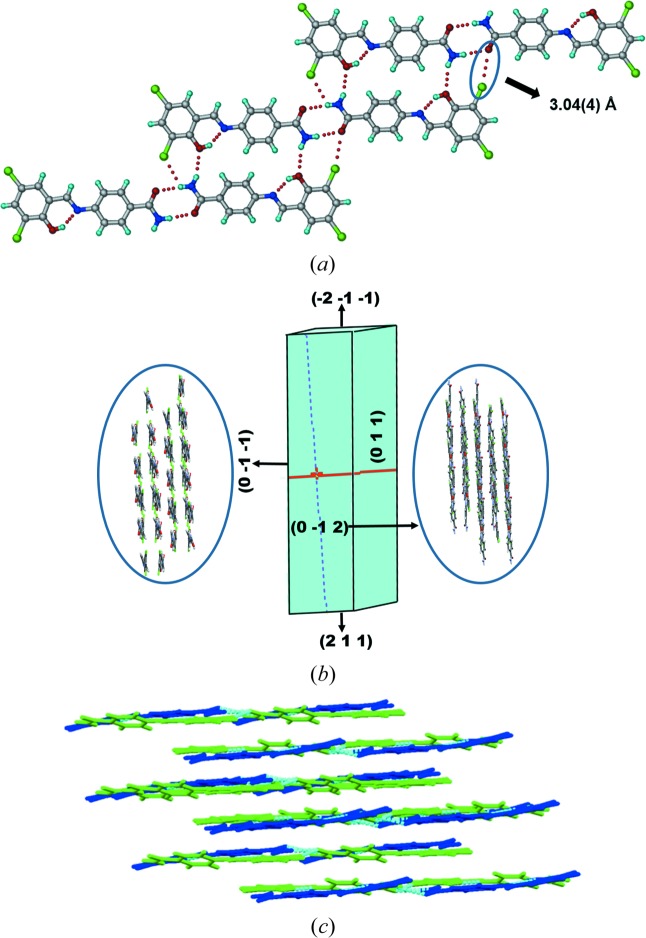
(*a*) Amide dimers extended by C—Cl⋯O and N—H⋯O interactions in Form III. (*b*) Morphology of Form III crystal and packing of molecules in different planes. (*c*) Layered arrangement of symmetry independent molecules (blue and green color).

**Figure 6:**
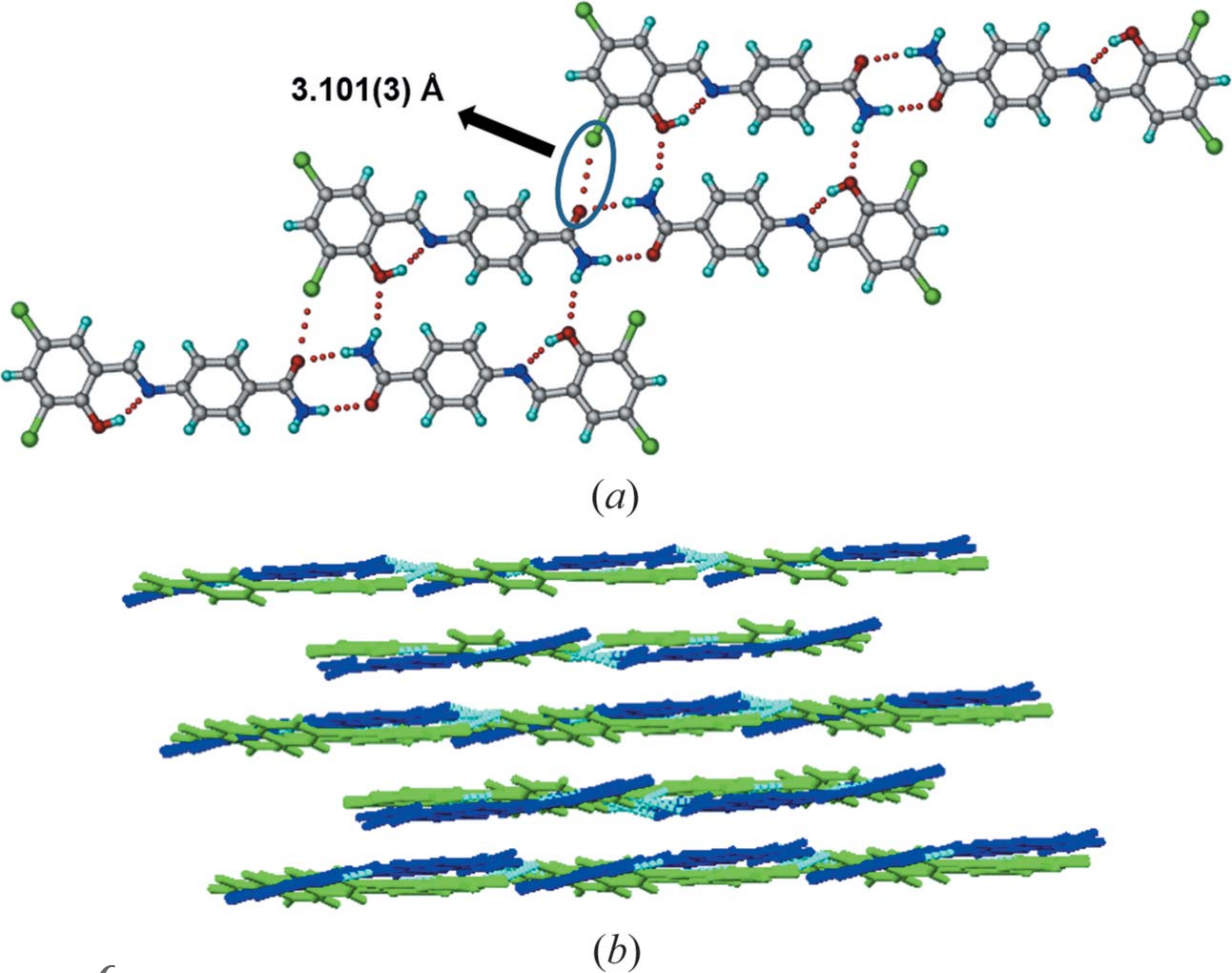
(*a*) Amide dimers extended by C—Cl⋯O and N—H⋯O interactions. (*b*) Layered arrangement of molecules (Form IV).

The X-ray crystal structures of Form I and II were solved in triclinic space group 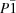. The amide group forms a dimer through N1—H1*B*⋯O1 [2.00 (4) Å, ∠172 (3)°] hydrogen bonds in a 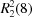 ring motif (Etter *et al.*, 1990 ▸; Bernstein *et al.*, 1995 ▸) in Form I (Fig. 3 ▸). An intramolecular hydrogen bond between the hydroxyl donor and imine nitrogen through the O2—H2*A*⋯N2 [1.66 (4) Å, ∠149 (4)°] ring makes an 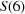 motif. The dihedral angle between the two phenyl rings is 20.07 (2)° (Table 1 ▸). The amide dimers extend through C—Cl⋯O [2.961 (3) Å] and N1—H1*A*⋯O2 [2.25 (4) Å, ∠161 (3)°] interactions in an eight-membered ring motif. The molecular layers are parallel to the crystallographic (1 −1 −2) plane at a distance of 3.4 Å between the molecular layers (Fig. 3 ▸). The crystal structure of Form II (Fig. 4 ▸) has the same amide dimer synthon [N1—H1*B*⋯O3; 2.06 (1) Å, ∠170 (1)° and N3—H3*B*⋯O1; 2.06 (3) Å, ∠170.4 (1)°] and N—H⋯O [N3—H3*A*⋯O2, 2.41 (2) Å, ∠144.3 (2)°; and N1—H1*A*⋯O4, 2.32 (4) Å, ∠157.4 (1)°] hydrogen bonds as in Form I. The number of symmetry independent molecules is different (*Z*′ = 1 in Form I and 2 in Form II). The dihedral angles between phenyl ring planes are 11.99 (17)° and 26.52 (17)°. The C—Cl⋯O interactions are 2.983 (2), 3.237 (2) Å. The chain grows parallel to the *c*-axis and the molecules are arranged in a wavy (corrugated) motif. Form III and IV also crystallized in the space group 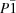 and with two molecules in the asymmetric unit. Forms III and IV (Figs. 5 ▸ and 6 ▸) have the same type of amide dimers which are described for the above two structures [N1—H1*B*⋯O3: 2.02 (2) Å, ∠170.7 (3)°; N3—H3*B*⋯O1: 2.17 (3) Å, ∠160.9 (1)°] and [N1—H1*B*⋯O3: 2.18 (2) Å, ∠161.2 (2)°; N3—H3*A*⋯O1: 2.04 Å, ∠170.8 (2)°], which extend *via* C—Cl⋯O interactions [3.04 (4), Fig. 5 ▸ *a*; and 3.101 (3) Å, Fig. 6 ▸ *a*]. The dihedral angle between two phenyl rings of the same molecule in Form III is 13.87 (4) and 23.91 (4)° and in Form IV is 12.72 (2) and 23.49 (2)°, respectively (Table 1 ▸). In Form III the amide dimers are connected by Cl⋯Cl interactions of Type I (Mukherjee *et al.*, 2014 ▸; Desiraju & Parthasarathy, 1989 ▸) [C—Cl⋯Cl–C, 3.466 (2) Å, ∠138.4 (2)°] to form a tetrameric ring motif (Fig. S2). The planar molecular layers are parallel to the crystallographic (101) plane in Form III (Fig. 5 ▸ *c*). The hydrogen-bond synthons in Forms I, II, III and IV are identical except that the Cl⋯O distances vary in the polymorphs (Table 2 ▸), but these slight differences in halogen-bond distances have a dramatic consequence on the thermal response of compound A crystals (for additional diagrams of Cl⋯Cl interactions and packing in Compound A polymorphs, see Figs. S2–S3), as described in the next section. The crystal structures and hydrogen-bonding interactions for compounds B and C are discussed in the supporting information  (Figs. S4–S7 and Table S3).

The powder X-ray diffraction lines of the three polymorphs of compound A are significantly distinct to permit characterization of the bulk material in each case (Fig. S8), as well as to monitor phase transitions. The peaks of Form I appear at 2θ 9.89, 10.84, 13.22, 13.66, 15.66°, for Form II at 7.08, 8.94, 11.58, 12.82, 14.19°, and Form III at 9.21, 12.32, 13.75°.

### 6.2. VT-PXRD and thermal analysis   

To better understand the events visually observed on the thermal microscope, variable-temperature powder X-ray diffraction (VT-PXRD) and DSC of the three polymorphs were performed. Heating the sample bench of XRD showed transformation to a new polymorph IV at 200°C for all the three polymorphs such as I, II and III. This transient Form IV was characterized by its unique powder XRD lines and in all cases the product on cooling to room temperature was Form III, which is the nearest stable phase (Fig. S9). Thus, a heat–cool cycle exhibited transformation to polymorph III *via* a new phase IV. In order to observe possible phase changes below ambient temperature (−25°C to 30°C), DSC was carried out on the sample in the range −150°C to 200 °C. Form I showed an endotherm at 147°C and saw tooth-like endotherms (zigzag profile) at 170–185°C due to Form I → IV conversion (confirmed by VT-PXRD experiments, Fig. S9). PXRD of high-temperature Form IV obtained from different polymorphs I–III are compared in Fig. S10. On cooling the same material, Form IV converted to Form III at 0–10°C and on reheating Form III, a small endotherm at 150–160°C was observed, after which it again converted to high-temperature phase Form IV (Form I → IV ↔ III is a solid-to-solid phase transformation); melting occurred at 249°C (Figs. S11 and S12). In the case of Form II, an endotherm was observed at 170°C and upon further heating a second endotherm occurred at 183–185°C, which indicates Form II → IV conversion. Reheating of the same material showed a small endotherm at 150–160°C (Form III → IV) followed by melting at 249°C (Figs. S13 and S14). During heating of Form III, a sawtooth wave DSC profile was observed at 181–190°C, indicating the conversion of Form III to IV. Cooling of the same material showed transformation to Form III, and on reheating small endotherms were observed at 150–162°C (Form III → IV), followed by melting at 251°C (Figs. S15 and S16). All the above experiments were performed multiple times with identical results. We also performed competitive slurry experiments to establish that Form II is more stable (thermodynamically stable state) compared with the remaining two Forms I and III. DSC and VT-PXRD confirmed that Form IV is stable at high temperature (after 180°C) and Forms III and IV are reversible during cooling and heating (III ↔ IV). Thus, the solid-to-solid phase transitions on temperature-modulated powder XRD and DSC exhibit similar transformations under heat–cool conditions. Compounds B and C melt at 274°C and 254 °C, respectively, without any phase transformation (Fig. S17).

### 6.3. Halogen bonds   

Compound A showed a mechanical response towards heating but not the Br and I derivatives, even though these structures have the same type of hydrogen bonds and halogen bonding synthons and are three-dimensional isostructural (*Xpac* analysis; Figs. S18–20). The most probable reason for mechanical response is mainly due to a change in the halogen atom and also the halogen-bond interactions (Table S3). The interaction energy for the C—*X*⋯O (*X* = F, Cl, Br and I) bond increases with an increase in polarizability and a decrease of electronegativity for the halogen atom (Politzer *et al.*, 2010 ▸; Riley & Hobza, 2008 ▸; Riley & Merz, 2007 ▸). The combined effects of both factors increases the σ hole for halogens towards a more +ve lobe in the order of I > Br > Cl. The increased electrostatic interaction between the σ hole of the halogen and oxygen lone pair electrons results in strengthening of the halogen bond from C—Cl⋯O through C—Br⋯O to C—I⋯O (Table 3 ▸) (Riley & Merz, 2007 ▸). In effect, the stronger halogen-bonded structures (having near-identical amide N—H⋯O hydrogen bonds) with Br and I atoms make them less responsive to temperature and mechanical stress because the halogen bond is too strong for the heavier halogens to show structural (and property) dynamics, indicating the importance of weaker C—Cl⋯O interactions in exhibiting the mechanical response of molecular crystals and temperature effects.

### 6.4. Thermosalient effect of molecular crystals   

During heating of Form I (Compound A) on the (1 0 1)/(−1 0 −1) faces (Fig. 3 ▸), we observed a mechanical response of the material by jumping (see the video in the supporting information). After heating Form I crystals for 5–10 min at 200°C, it converted to Form IV with a small change in unit-cell volume (Δ*V* = 20 Å^3^), after correcting for *Z*′ being 1 and 2 in Form I and IV, respectively. Similarly Forms II and III also converted to Form IV after heating for 5 min with a much larger change in volume (Δ*V* = 64, 41 Å^3^). Examination of the individual cell axes (Table 4 ▸) suggests that there is a decrease in the cell length along the *a*-axis and increase in the *b*- and *c*-direction of the triclinic cells of Forms I and II. Form III crystals also showed jumping (see the video in the supporting information) when heated on the (0 1 1)/(0 −1 −1) faces with a slight increase in cell lengths in *a*, *b* and *c* directions. In contrast, Form II crystals have irregular morphology (Table S2), so we were not able to identify specific faces in which the crystal shows a response to heating. Heating Form II crystals beyond 180°C caused blasting (see the video in the supporting information). To understand the reason behind the different mechanical responses (jumping and blasting) of polymorphic molecular crystals to heating, we compared all four polymorphic structures in terms of variation in conformation, hydrogen-bonding interactions and changes in crystal packing (Table S2). During the transition all hydrogen/halogen bonds were retained in the structure with slight changes in the distances along with slight changes in conformation in the molecule, and significant changes in packing of molecules were observed. Forms I and III molecules are arranged in a layered structure (Figs. 3 ▸ and 5 ▸), whereas in form II symmetry-independent molecules are arranged in a corrugated wave motif (Fig. 4 ▸). We hypothesize that upon heating crystals of Form I and Form III, the heat is transferred uniformly from the face resulting in the transmission of thermal stress largely in a single direction, thereby causing a thermosalient effect of jumping. In Form II, however, due to the corrugated wave-like arrangement of molecules, heat transmission is non-uniform resulting in a sudden blast of the crystal. The most likely explanation for these effects is due to the sudden release of accumulated strain energy during phase transition (Etter & Siedle, 1983 ▸), and anisotropy in the cell parameters. However, such thermosalient effects in molecular crystals is still not fully understood due to the limited number of examples in the literature for structure–property correlation. Thermochromic effects in Compound A polymorphs are described in the supporting information (Fig. S21).

**Figure 7:**
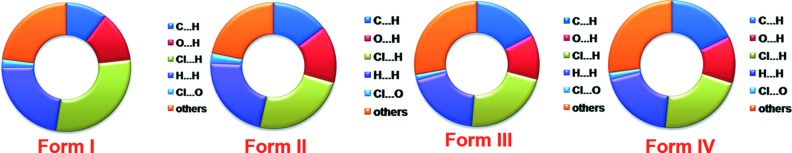
Percentage contribution of hydrogen-/halogen-bonding interactions in Compound A polymorphs (I–IV).

Hirshfeld surface (Poulsen *et al.*, 2009 ▸) analysis of polymorphs of compound A (Fig. 7 ▸) showed that Form II has a greater contribution from O⋯H and Cl⋯O contacts (14.8 and 2.6%) compared with Form I (12.9 and 2.1%), Form III (11.8 and 1.5%) and Form IV (12.1 and 1.8%, Fig. S22) indicating the stability of Form II compared with the other two forms, which was also supported in competitive slurry experiments.

## 7. Conclusion   

We have described thermal responses of jumping (Forms I and III) and blasting (Form II) in dichloro *N*-salicylidene (compound A) polymorphs due to the sudden phase transition at high temperature. The bromo and iodo halogen derivatives did not exhibit any thermal responses. The significance of weaker Cl⋯O interactions in an invariant amide dimer N—H⋯O structural family of polymorphs is the reason ascribed to the mechanical response of chloromolecular crystals. This study demonstrates the utility of hydrogen- and halogen-bonded molecular crystals in exhibiting the thermosalient effect under thermal stress and also presents a rationale for the design of thermoresponsive crystals.

## 8. Related literature   

The following references are cited in the supporting information for this article: Cohen *et al.* (1964 ▸), Hadjoudis & Mavridis (2004 ▸), Haneda *et al.* (2007 ▸), Hutchins *et al.* (2014 ▸) and Senier & Shepheard (1909 ▸).

## Supplementary Material

Crystal structure: contains datablock(s) AFORMI, AFORMII, AFORMIII, FormIV, compoundB, CompoundC. DOI: 10.1107/S2052252517004043/lq5003sup1.cif

Structure factors: contains datablock(s) AFORMI. DOI: 10.1107/S2052252517004043/lq5003AFORMIsup2.hkl

Structure factors: contains datablock(s) AFORMII. DOI: 10.1107/S2052252517004043/lq5003AFORMIIsup3.hkl

Structure factors: contains datablock(s) AFORMIII. DOI: 10.1107/S2052252517004043/lq5003AFORMIIIsup4.hkl

Structure factors: contains datablock(s) FormIV. DOI: 10.1107/S2052252517004043/lq5003FormIVsup5.hkl

Structure factors: contains datablock(s) compoundB. DOI: 10.1107/S2052252517004043/lq5003compoundBsup6.hkl

Structure factors: contains datablock(s) CompoundC. DOI: 10.1107/S2052252517004043/lq5003CompoundCsup7.hkl

Supporting figures and tables. DOI: 10.1107/S2052252517004043/lq5003sup8.pdf

CCDC references: 1488992, 1488993, 1488994, 1524448, 1488995, 1488996

## Figures and Tables

**Table 1:** Dihedral angle between the two phenyl rings in crystal structures

Compound	Dihedral angle (in °)
Compound A Form I	20.07
Compound A Form II	11.99, 26.52
Compound A Form III	13.87, 23.91
Compound A Form IV	12.72, 23.49
Compound B	18.08
Compound C	13.48

**Table 2:** Cl⋯O distances in polymorphs of compound A

Polymorph	C—Cl⋯O (Å)
Form I	2.961 (3)
Form II	2.983 (2), 3.237 (2)
Form III	3.04 (4)
Form IV	3.101 (3)

**Table 3:** Energy of halogen bonds (see Table S4)

Halogen bond (C—*X*⋯O)	Stabilization energy (kcal mol^−1^)
C—Cl⋯O (Form I)	−0.07
C—Br⋯O	−0.52
C—I⋯O	−1.70

**Table 4:** Cell parameters of polymorphs of Compound A

Polymorph	Form I (at 25°C)	Form II (at 25°C)	Form III (at 25°C)	Form IV (at 180°C)
*a*	8.44	8.35	7.27	7.43
*b*	9.11	12.75	13.27	13.29
*c*	9.15	13.01	14.54	14.69
*V* (Å^3^)	673.27	1322.2	1345.3	1386.5
Density	1.525	1.553	1.526	1.481
