# Supplementary material for: Mechanochemical synthesis of N-salicylidene­aniline: thermosalient effect of polymorphic crystals
Source: IUCrJ. 2017 Mar 27;4(Pt 3):243–50. doi: 10.1107/S2052252517004043 (PMC5414398; doi:10.1107/S2052252517004043)
Supplement: Supplementary file 8 [file m-04-00243-sup8.pdf]

IUCrJ

Volume 4 (2017)

Supporting information for article:

Mechanochemical synthesis of *N*-salicylideneaniline: thermosalient effect of polymorphic crystals

Sudhir Mittapalli, D. Sravanakumar Perumalla and Ashwini Nangia

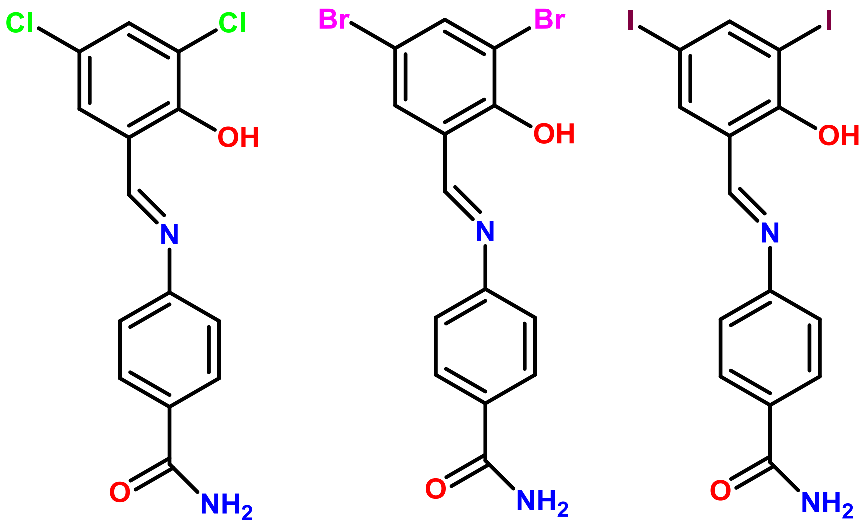

A = (E)-4-(3,5-Dichloro-2-hydroxybenzylidene)amino)benzamide.

B = (E)-4-(3,5-Dibromo-2-yydroxybenzylidene)amino)benzamide.

C = (E)-4-(3,5-Diiodo-2-hydroxybenzylidene)amino)benzamide.

Figure S1 Chemical structures of the compound synthesized in present work.

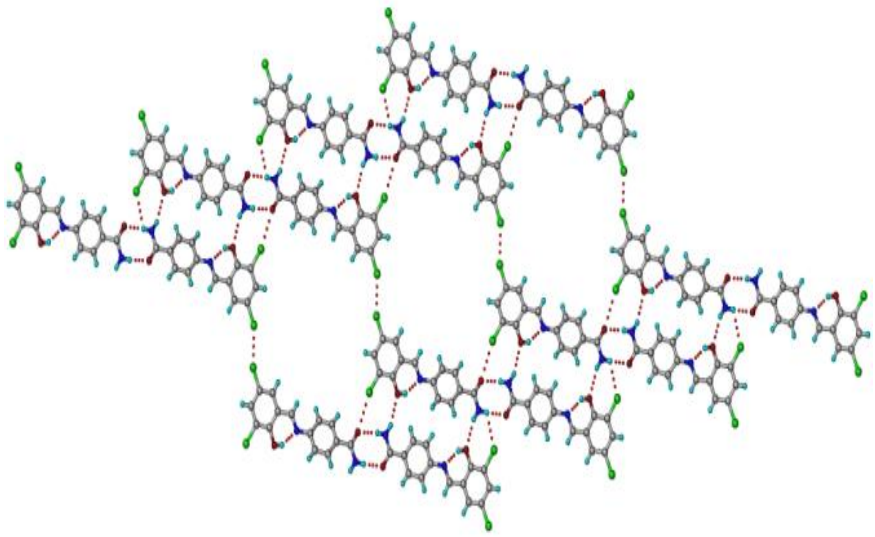

Figure S2 Amide dimers are connected by Cl···Cl (type I) interactions in the crystal structure of Form III of compound-A.

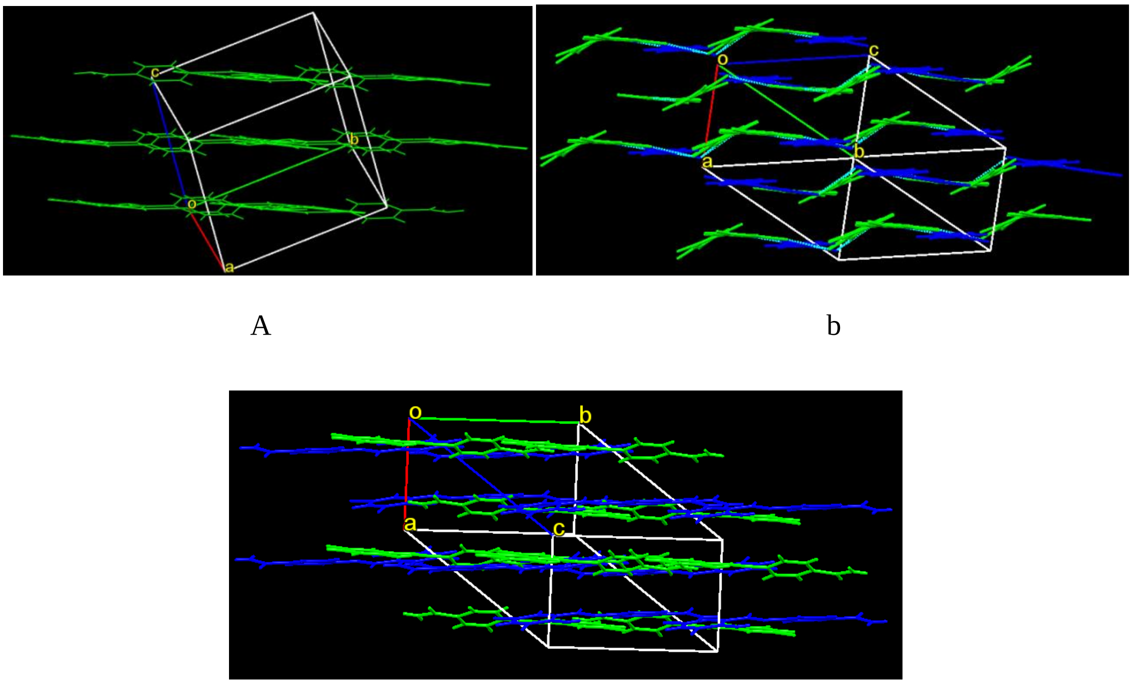

Figure S3 Projection of the Compound-A Form I crystal packing along (1 -1 -2) plane (a), Form II (b), and Form III crystal packing along (1 0 1) plane (c).

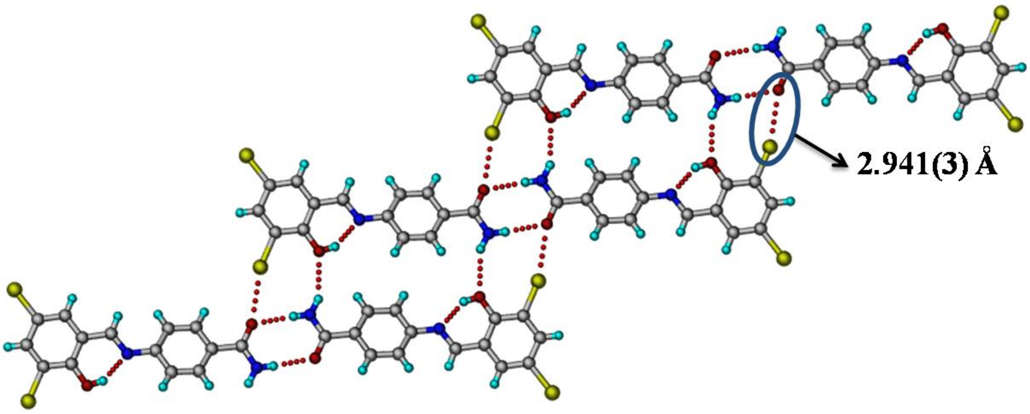

Figure S4 Amide N–H···O dimers extend via C–Br···O interactions in the crystal structure of compound-B.

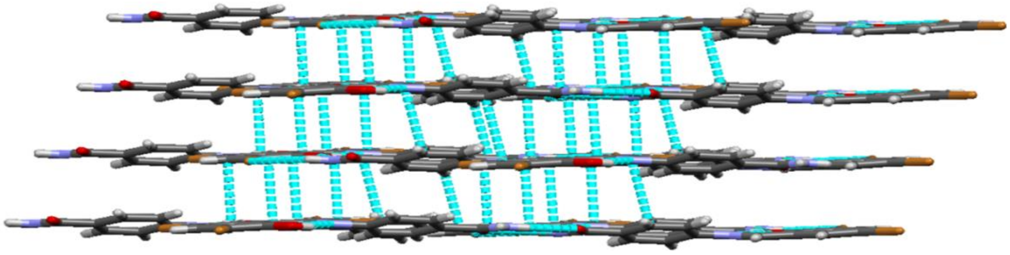

Figure S5 The layered arrangement of molecules in compound-B.

Compound-B was crystallized and its crystal structure was solved in triclinic space group *P*-1. The amide group forms a dimer through N1–H1B···O1 (2.12 Å, ∠175°; Figure S4) H bonds in $R_2^2(8)$ ring motif. [1] An intramolecular hydrogen bond is present between the hydroxy group and imine nitrogen through O2–H2A···N2 (1.82 Å, ∠148°) bond in S(6) motif. The layered arrangement of molecules is shown in Figure S5.

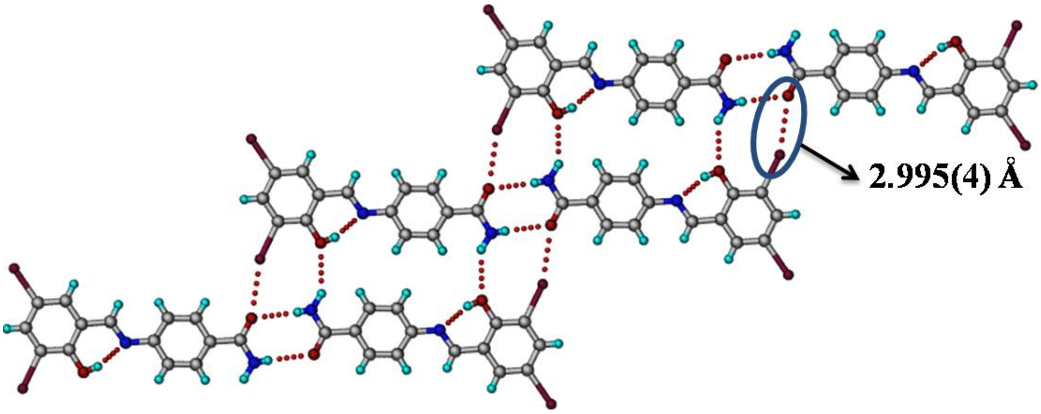

Figure S6 Amide N–H···O dimers extend through C–I···O interactions in the crystal structure of compound-C.

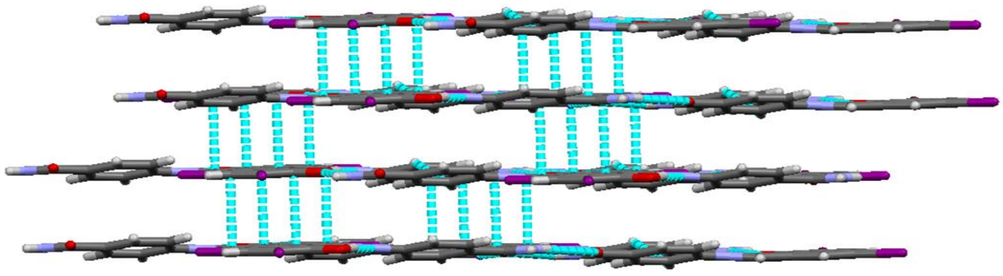

Figure S7 Showing π-stacking interactions between the molecular layers in compound-C.

The crystal structure of compound-C was solved in triclinic space group *P*-1. Amide group forms a dimer through N1–H1B···O1 (2.18 Å, ∠177°; Figure S6) H bonds in $R_2^2(8)$ ring motif. An intramolecular hydrogen bond was observed between hydroxy group and imine nitrogen through O2–H2A···N2 (1.84 Å, ∠159°) bond S(6) motif. The layered structure is shown in Figure S7.

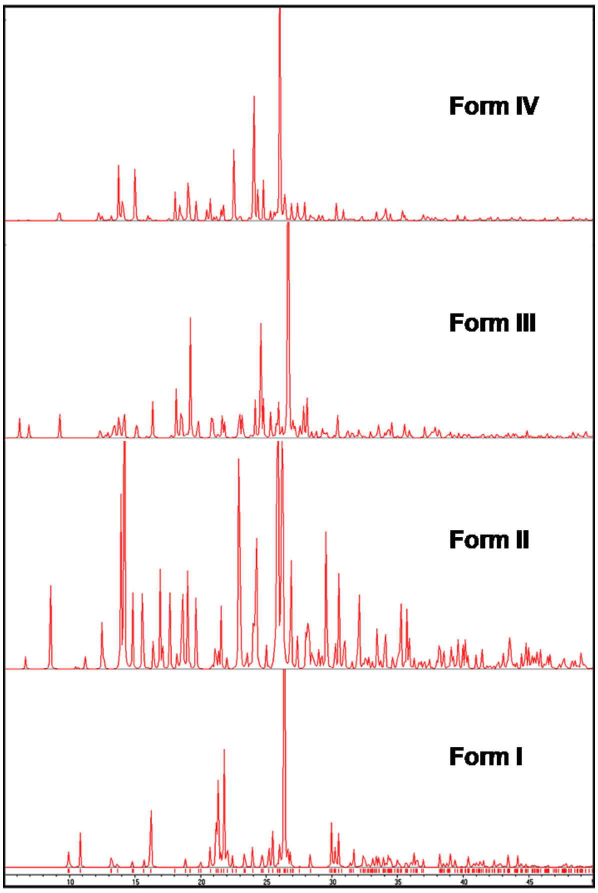

Figure S8 Overlay of powder XRD pattern of Form I, II, III and IV of compound-A.

The powder X-ray diffraction lines of the four polymorphs of compound-A are significantly distinct to permit characterization of the bulk material in each case (Figure S8), as well as to monitor phase transitions. The peaks of Form I appear at 2θ 9.89, 10.84, 13.15, 13.66, 15.66, 16.19°, for Form II at 7.08, 8.94, 11.58, 12.82, 14.19, 14.5°, for Form III at 6.15, 6.86, 9.21, 12.32, 13.75, 14.95° and Form IV at 9.18, 12.22, 13.15, 13.73, 14.03, 14.97, 22.52, 26.0°.

S1. Modulated Temperature Powder XRD

Powder XRD was recorded on the three polymorphs of compound-A to verify the phase homogeneity of the bulk material and the differences in their signature peaks. Upon heating Form I at 200 °C for 45 min, it converted to a new Form IV. On cooling of the same material Form IV converted to Form III. Heating Form II for 40-45 min showed conversion to Form IV which on cooling again converted to Form III. Heating Form III at 190-200 °C for about 5-10 min (shorter time compared to Form-I and Form-II) exhibited changes in the powder pattern indicating the new Form IV, which on cooling again converted to Form III. We performed cyclic heating and cooling experiments on Form III, which showed that Form III and Form IV inter-conversions are reversible and that Form IV is stable at high temperature only (Figure S9). The X-ray crystal structure of this new Form IV, a high T phase at 200 °C could not be obtained because it reverts to Form III on cooling to RT.

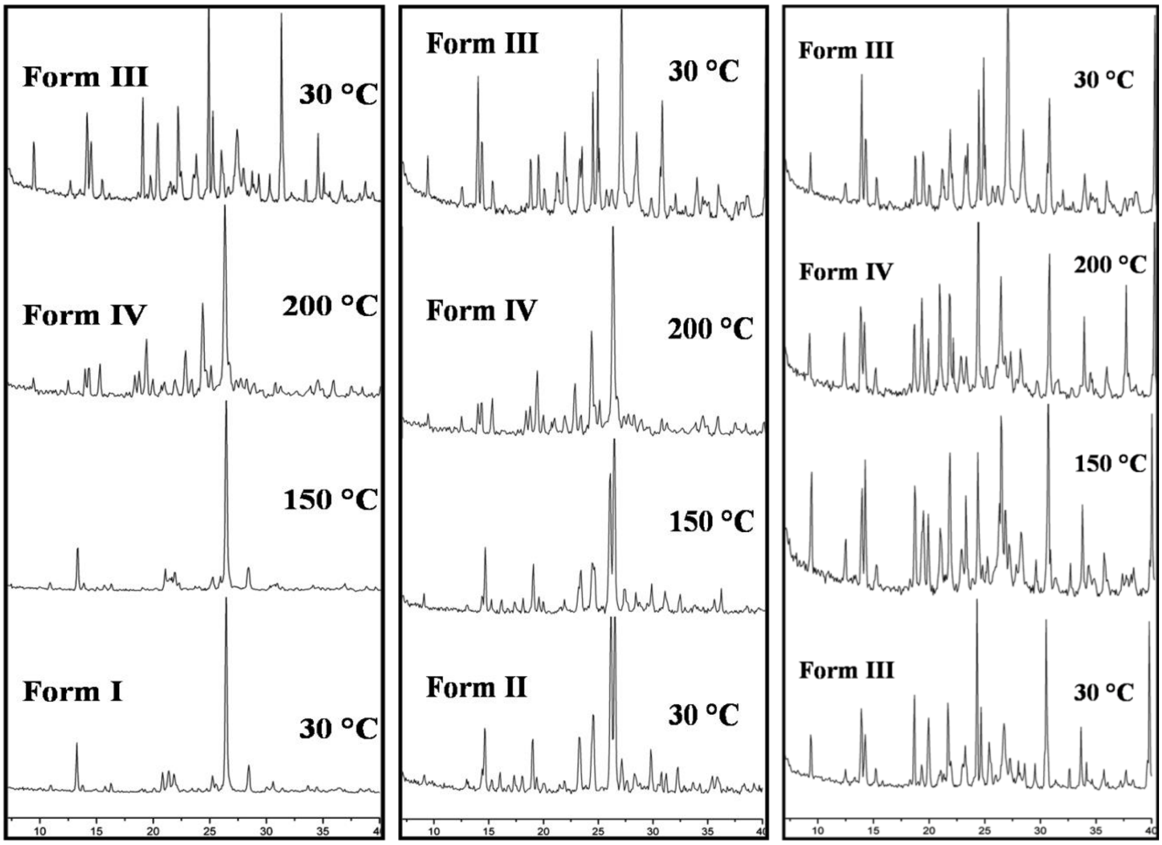

A Form I **A Form II** **A Form III**

Figure S9 Overlay of Powder patterns of Form I, II and III of compound-A at different temperatures. Since the duration of heating is much longer for polymorph I and II (45 min) compared to polymorph III (5 min), the crystalline quality of the product is different in that several of the high 2θ peaks are absent in the PXRD of high T Form IV in the left and middle panels. This could be due to loss in crystallinity due to prolonged heating.

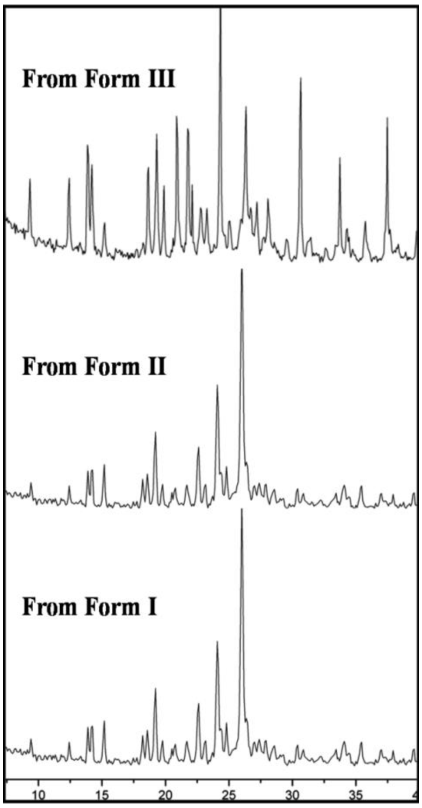

Figure S10 Overlay of Form IV (high temperature polymorph) obtained by heating Form I, II and III in separate experiments is shown to compare the diffraction peak profiles. The additional peaks at high 2θ (>27°) in Form IV powder XRD obtained from Form III because this transformation requires minimal heating of 5 min (higher crystallinity) compared to 45 min for Form I and II.

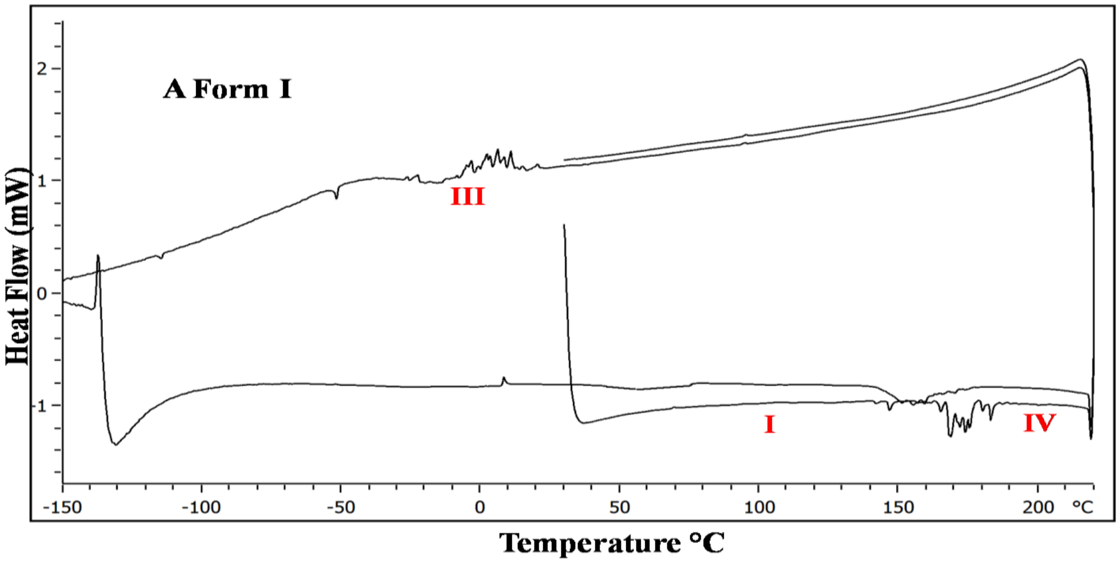

Figure S11 DSC thermogram of compound-A Form I (heat-cool-reheat cycle).

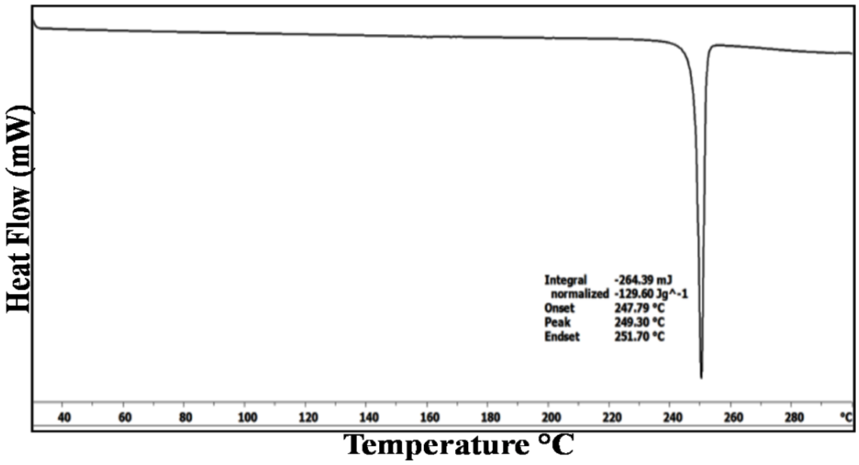

Figure S12 DSC thermogram of compound-A Form I (melting).

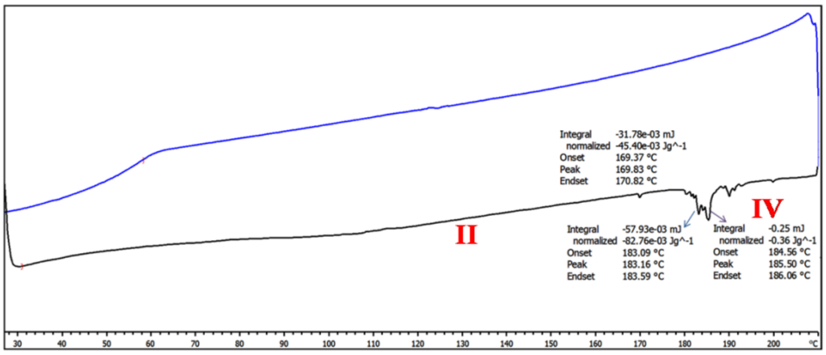

Figure S13 DSC thermogram of compound-A Form II (heat-cool cycle).

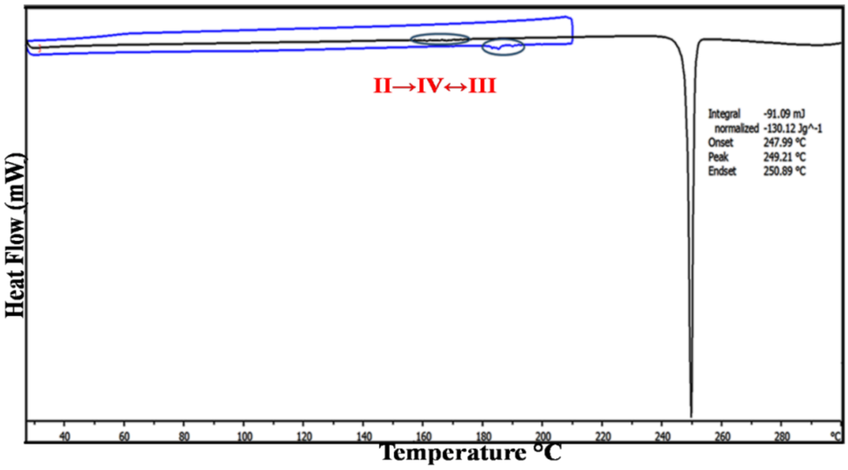

Figure S14 DSC thermogram of compound-A Form II (heat-cool (blue) and reheat (black)).

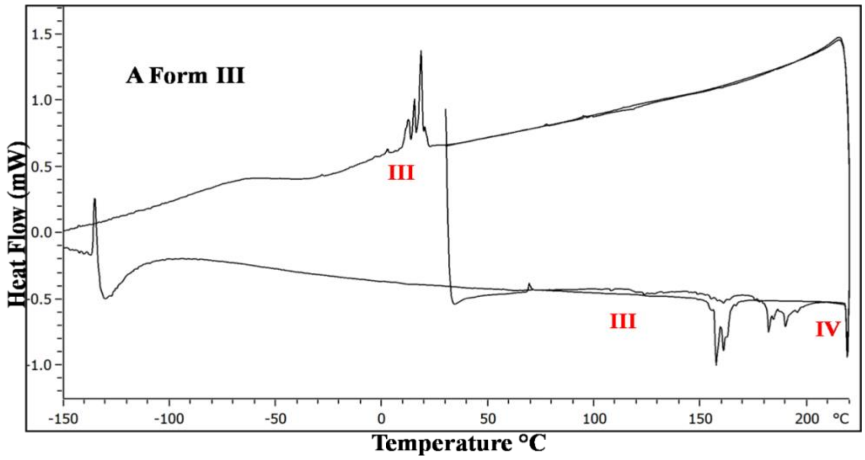

Figure S15 DSC thermogram of compound-A Form III (heat-cool cycle).

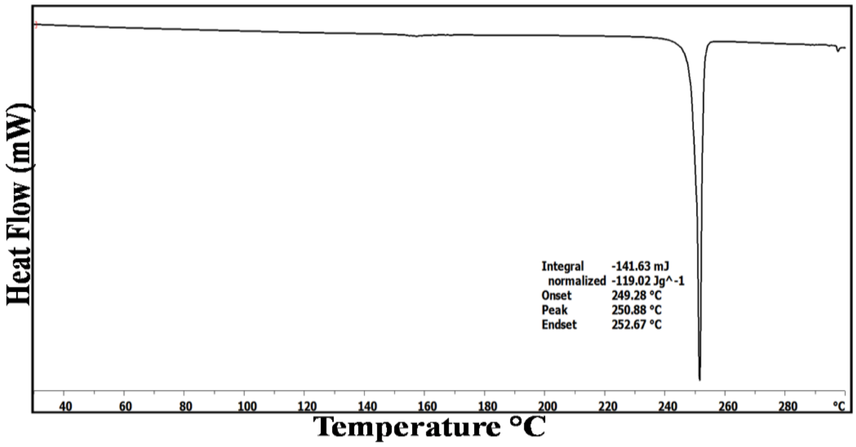

Figure S16 DSC thermogram of compound-A Form III (melting).

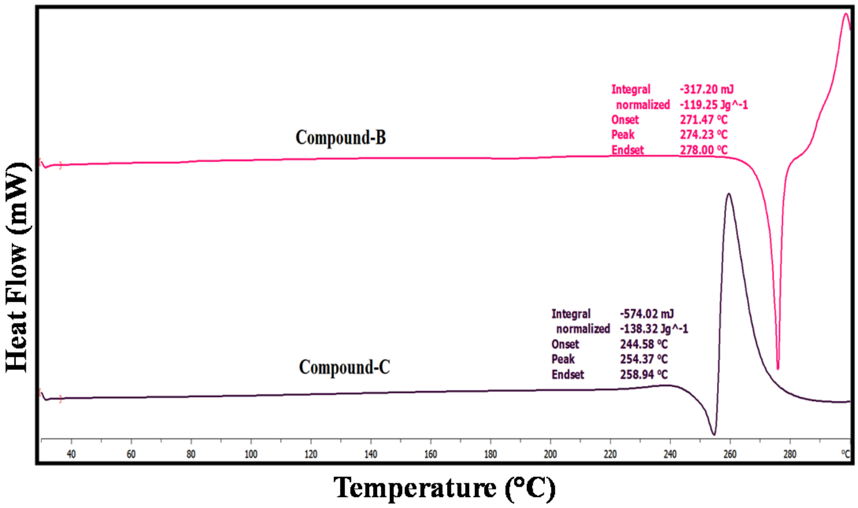

Figure S17 DSC thermogram of compound-B and C.

S2. X Pac Analysis

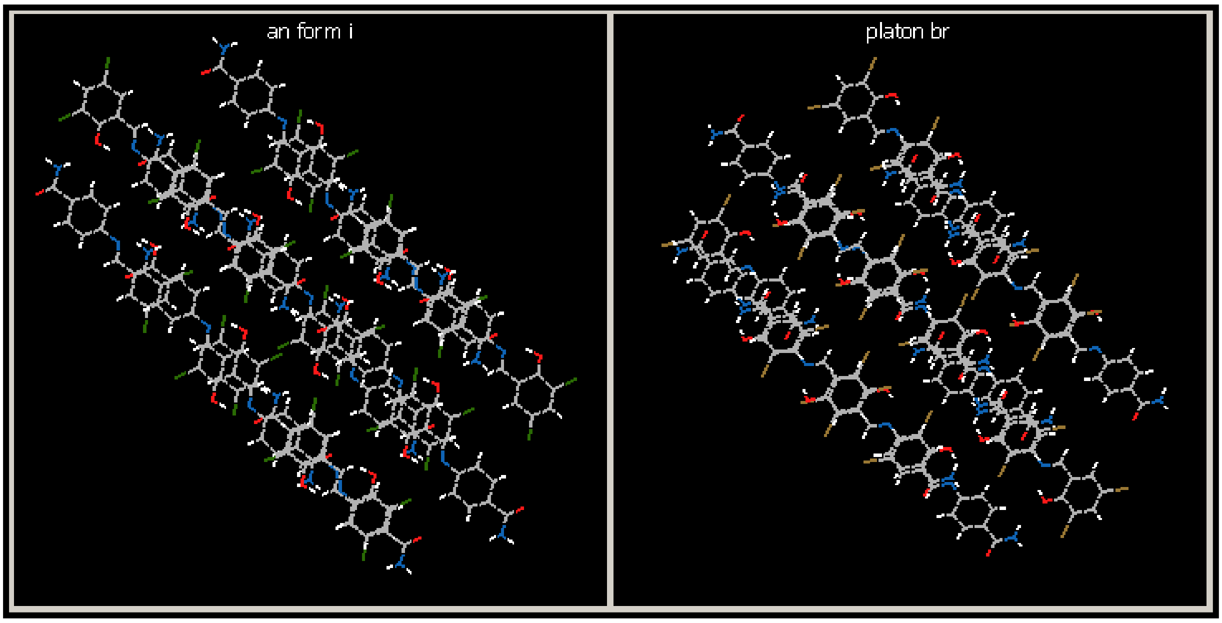

a

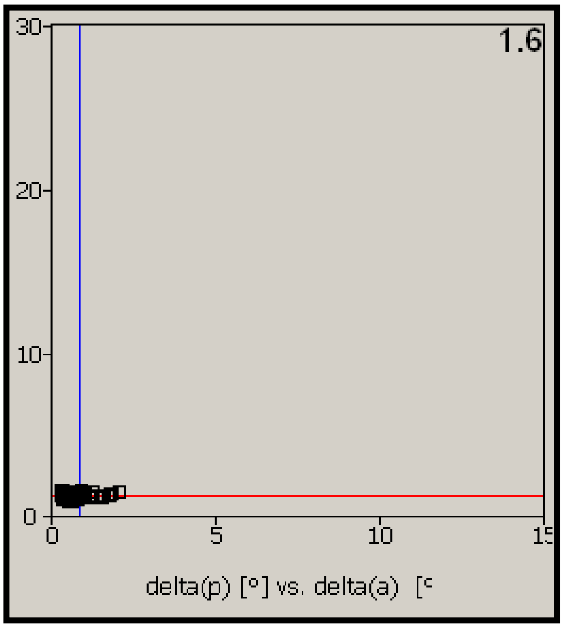

b

Figure S18 (a) XPac analysis of compound-A (Form-I) and compound-B. 16 molecules are identically arranged in the supramolecular construct. (b) The interplanar angular deviation (δp, x-axis) vs. angular deviation (δa, y-axis) plot (in °) gives a dissimilarity index of 1.6, indicative of 3D isostructurality.

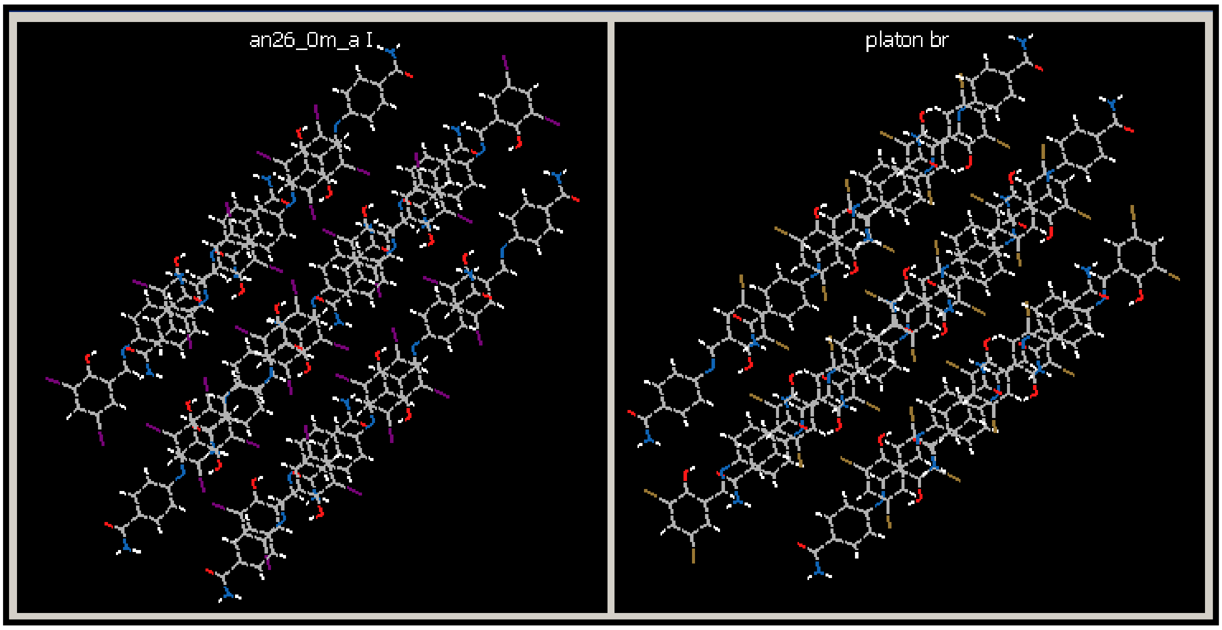

a

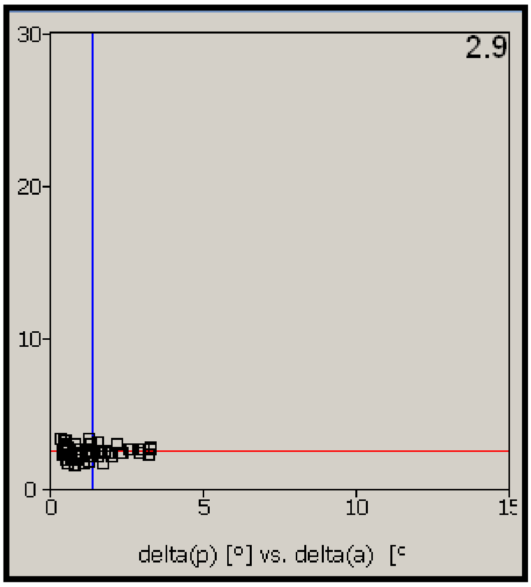

b

Figure S19 (a) XPac analysis of compound-B and compound-C, 16 molecules are identically arranged in the supramolecular construct. (b) The interplanar angular deviation (δp, x-axis) vs. angular deviation (δa, y-axis) plot (in °) indicates a dissimilarity index of 2.9 due to the 3D isostructurality.

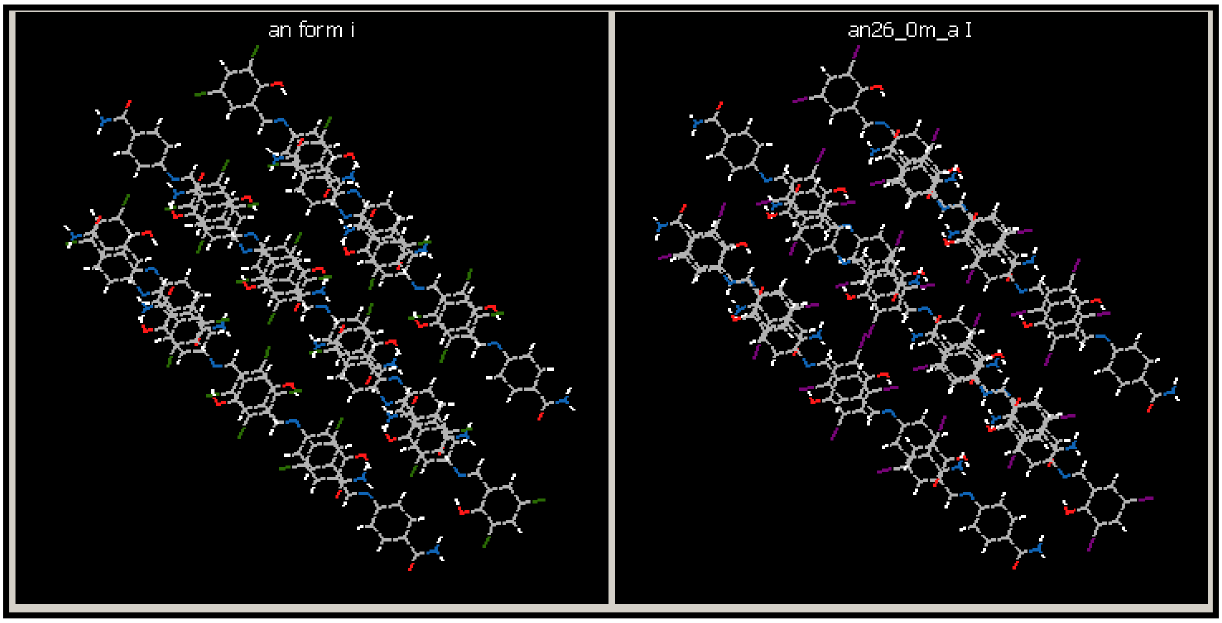

a

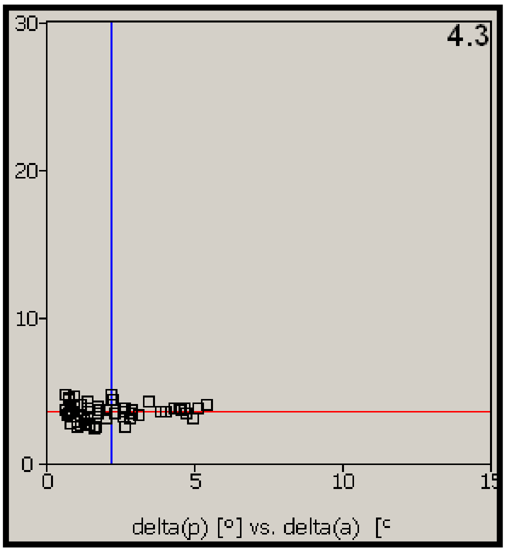

b

Figure S20 (a) XPac analysis of the compound-C and compound-A (Form-I), 16 molecules are identically arranged in the supramolecular construct. (b) The interplanar angular deviation (δp, x-axis) vs. angular deviation (δa, y-axis) plot (in °) indicates a dissimilarity index of 4.3 due to the 3Disostructurality.

S2. Thermochromism

Thermochromism is the reversible color change property of a substance in the solid-state with respect to temperature. The color change of salicylideneanilines is due to proton-transfer from enol to imine nitrogen (Figure S21) (Ogawa et al., 1998). The increase in the population of cis-keto form (Cohen et al., 1964; Senier et al., 1909; Hadjoudis et al., 2004) along with planarity of the structure are responsible

for thermochromic behaviour. Crystals are expected to be thermochromic when the dihedral angle between the phenyl rings ϕ< 25°, and photochromic when ϕ>25° (Haneda et al., 2007; Hutchins et al., 2014). In the crystalline state, the molecules are slightly away from planarity (Table 4) and have an intramolecular hydrogen bond between the enol proton and the imine nitrogen. At room temperature the color of the compound is red, on heating the material turns to thick red, and on crash cooling the color changes to yellow.

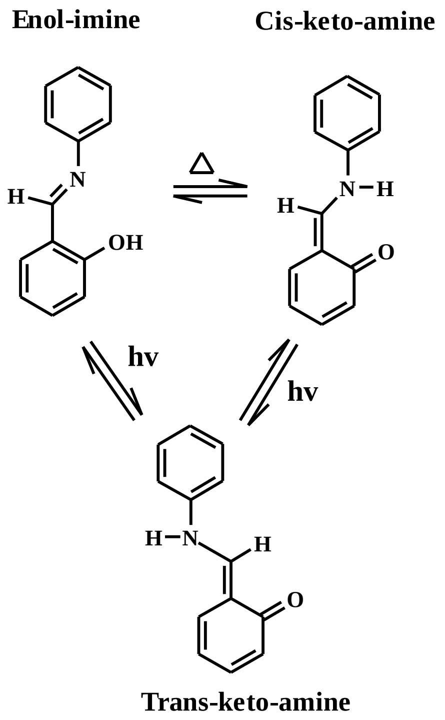

Figure S21 Reversible thermochromic and photochromic process in Salicylidene anilines.

S3. Hirshfeld Surface Analysis

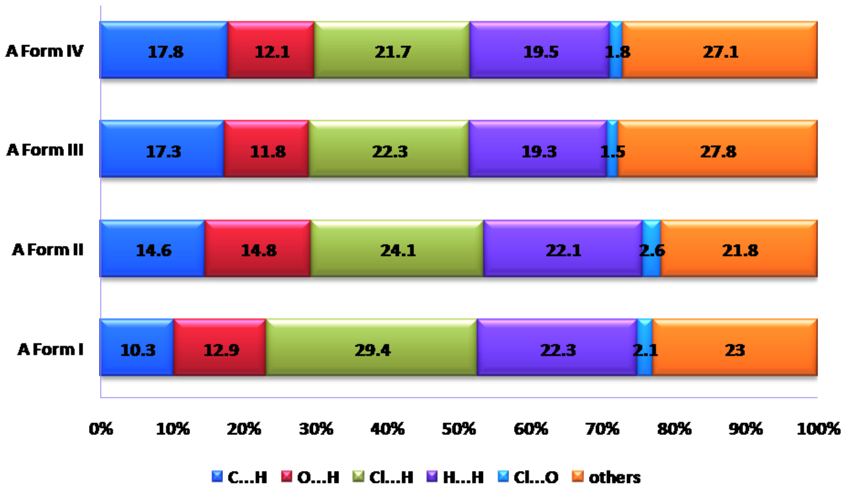

S3.1. Compound-A Form I

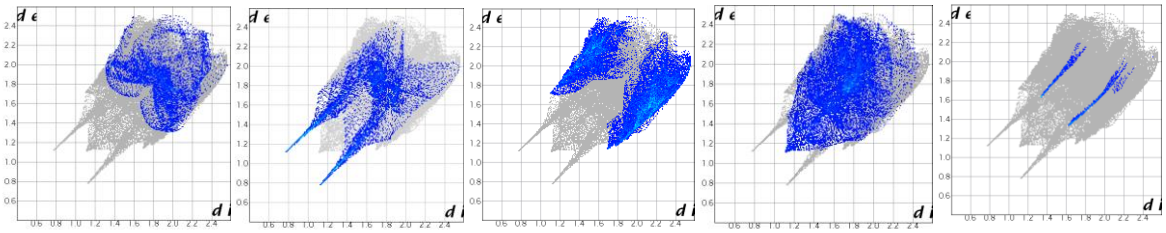

C…H 10.3 % O…H 12.9 % Cl…H 29.4 % H…H 22.3 % Cl…O 2.1 %

S3.2. Compound-A Form II

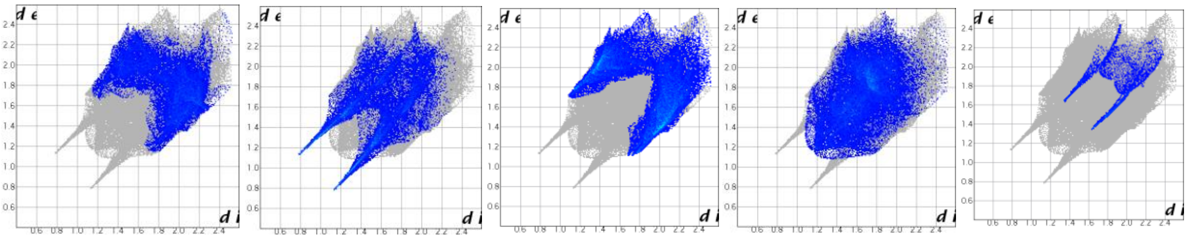

C…H 14.6 % O…H 14.8 % Cl…H 24.1 % H…H 22.1 % Cl…O 2.6 %

S3.3. Compound-A Form III

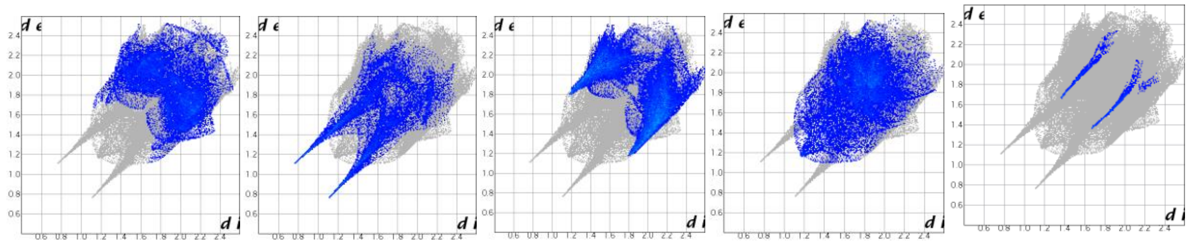

C…H 17.3 % O…H 11.8 % Cl…H 23.3 % H…H 19.3 % Cl…O 1.5 %

S3.4. Compound-A Form IV

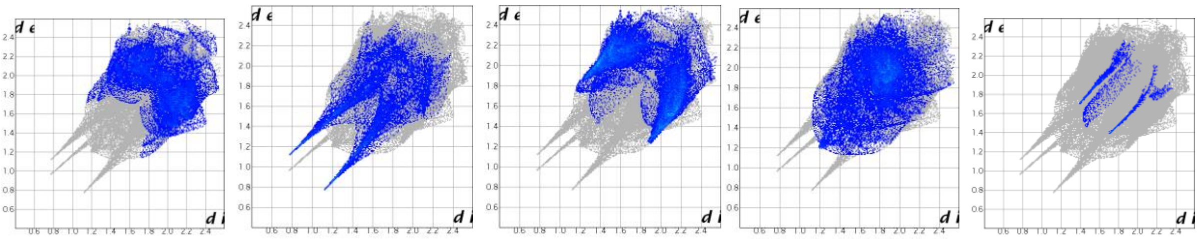

C…H 17.8 % O…H 12.1 % Cl…H 21.7 % H…H 19.5 % Cl…O 1.8 %

Figure S22 Hirshfeld surface analysis of polymorphic forms for compound-A.

Table S1 Crystallographic data.

	Compound A Form I	Compound A Form II	Compound A Form III	Compound A Form IV	Compound B	Compound C
Empirical Formula	$C_{14}H_{10}Cl_2N_2O_2$	$C_{14}H_{10}Cl_2N_2O_2$	$C_{14}H_{10}Cl_2N_2O_2$	$C_{14}H_{10}Cl_2N_2O_2$	$C_{14}H_{10}Br_2N_2O_2$	$C_{14}H_{10}I_2N_2O_2$
Formula weight	309.14	309.14	309.14	309.14	398.06	492.04
Crystal system	Triclinic	Triclinic	Triclinic	Triclinic	Triclinic	Triclinic
Space group	*P*-1	*P*-1	*P*-1	*P*-1	*P*-1	*P*-1
T (K)	298	298	298	453	298	298
a (Å)	8.449(7)	8.355(4)	7.2798(9)	7.4309(14)	8.3928(7)	8.4678(6)
b (Å)	9.116(5)	12.751(9)	13.2794(15)	13.296(2)	9.2815(8)	9.6066(7)
c (Å)	9.154(8)	13.013(9)	14.5473(9)	14.694(3)	9.3877(8)	9.6522(7)
α (deg)	81.461(6)	78.050(6)	87.618(7)	87.60(6)	81.620(7)	81.239(2)
β (deg)	77.033(7)	77.167(5)	80.816(7)	80.034(7)	75.049(7)	84.450(2)
γ (deg)	80.551(6)	88.734(4)	75.707(10)	75.859(7)	81.589(7)	72.336(2)
V (Å^3)	673.27(9)	1322.24(14)	1345.3(2)	1386.5(4)	694.45(11)	738.36(9)
D_{calcd} (gcm^{-3})	1.525	1.553	1.526	1.481	1.904	2.213
μ (mm^{-1})	4.366	4.447	4.371	0.470	7.436	4.261
θ range	4.91 to 66.6	3.54 to 66.59	4.60 to 66.59	2.868 to 25.026	4.84 to 66.60	2.24 to 27.60
Z/Z'	2/1	4/2	4/2	4/2	2/1	2/1
Range *h*	–10 to 10	–6 to 9	–8 to 8	–8 to 8	–9 to 8	–11 to 11
Range *k*	–10 to 10	–15 to 15	–12 to 15	–15 to 15	–10 to 11	–12 to 12
Range *l*	–10 to 10	–15 to 15	–17 to 16	–17 to 17	–11 to 8	–12 to 12
Reflections collected	5392	8563	8177	38263	3838	32730
Total reflections	2366	4668	4683	4568	2433	3409
Observed reflections	1385	3663	2627	3781	2179	2675
R_1 [$I > 2\sigma(I)$]	0.049	0.043	0.061	0.0541	0.036	0.033
wR_2 (all)	0.118	0.119	0.217	0.1472	0.100	0.081

Goodness-of-fit	0.977	1.042	0.988	1.044	1.050	1.017
X-ray diffractometer	OXFORD Xcalibur	OXFORD Xcalibur	OXFORD Xcalibur	Bruker D8 venture	OXFORD Xcalibur	Bruker D8 Quest

Table S2 Morphology of crystals and their faces for compound-A.

Form I		
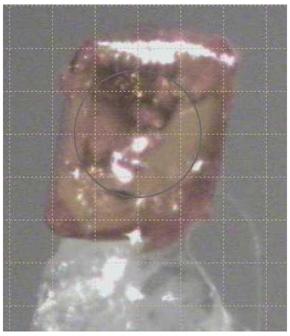	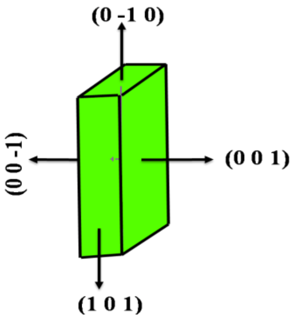	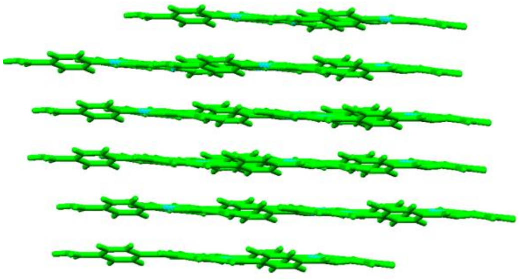<br>**Layered arrangement**
**Form III**		
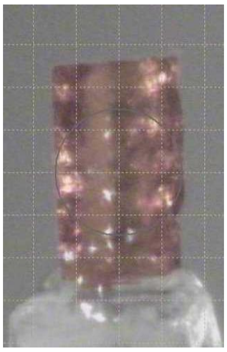	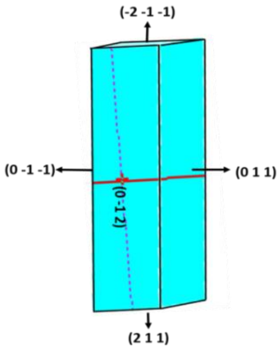	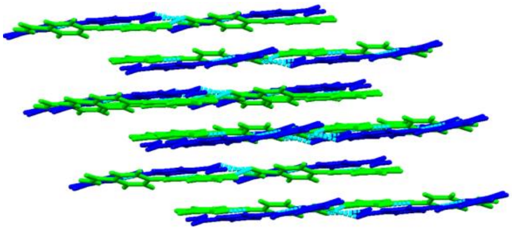<br>**Layered arrangement**
**Form II**		
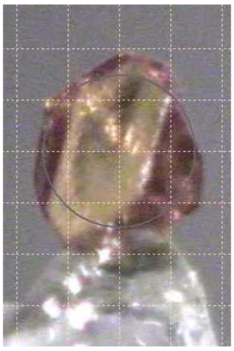	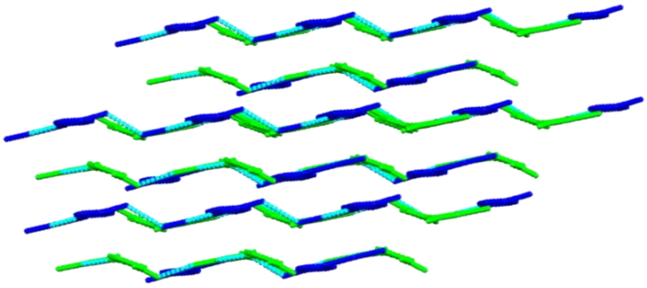<br>**Corrugated Wave**	

Table S3 Hydrogen bond geometry (neutron-normalized D–H distance).

	Interaction	D–H/Å	H···A/Å	D···A/Å	∠D–H···A/Å	Symmetry code
**Compound-A Form I**	N1–H1B···O1	0.91(4)	2.00(4)	2.905(5)	172(3)	1–x,–1–y,1–z
	O2–H2A···N2	0.99(4)	1.66(4)	2.556(4)	149(4)	Intramolecular
	N1–H1A···O2	0.92(4)	2.25(4)	3.131(4)	161(3)	2–x,–y,1–z
	C13–Cl2···O1			2.961(3)	166.2(1)	Intermolecular
**Compound-A Form II**	N1–H1B···O3	0.86	2.06	2.911(3)	170(1)	–1+x,y,–1+z
	N1–H1A···O4	0.86	2.32(4)	3.136(3)	157.4(1)	–1+x,y,z
	O2–H2A···N2	0.82	1.81	2.541(3)	148	Intramolecular
	N3–H3B···O1	0.86	2.06(3)	2.914(3)	170.4(1)	1+x,y,1+z
	N3–H3A···O2	0.86	2.41(2)	3.144(3)	144.3(2)	1+x,y,z
	C27–Cl4···O3			2.983(2)	165.9(1)	Intermolecular
	C13–Cl2···O1			3.237(2)	136.4(1)	Intermolecular
**Compound-A Form III**	N1–H1A···O4	0.86	2.31	3.129(5)	158	
	N1–H1B···O3	0.86	2.02	2.872(6)	170.7(3)	x,1+y,z
	O2–H2A···N2	0.82	1.81	2.545(5)	148	Intramolecular
	N3–H3A···O2	0.86	2.29	3.089(5)	154	x, y, z
	N3–H3B···O1	0.86	2.17(3)	2.993(6)	160.9(1)	x,–1+y,z
	O4–H4A···N4	0.82	1.82	2.548(5)	147	Intramolecular
	C27–Cl4···O3			3.040(4)	163.6(2)	Intermolecular
	C26–Cl3···Cl3			3.466(2)	138.4(2)	Intermolecular
**Compound-A Form IV**	N1–H1A···O4	0.86	2.33	3.128(3)	154	x, y, z
	N1–H1B···O3	0.86	2.18(2)	3.009(3)	161.2(2)	x,-1+y,z
	O2–H2A···N2	0.90	1.74	2.560(3)	151(4)	Intramolecular
	N3–H3A···O1	0.86	2.04	2.893(3)	170.8(2)	x,1+y,z
	N3–H3B···O2	0.86	2.36	3.173(3)	157.1	x, y, z
	C13–Cl2···O1			3.101(3)	163(1)	Intermolecular
	N3–H1B···O1	0.82(6)	2.12(5)	2.941(4)	175(5)	x, y, z
	N1–H1A···O2	0.88(7)	2.39(6)	3.204(5)	153(5)	x, y, z

**Compound-B**	O2–H2A···N2	0.82(4)	1.82(4)	2.557(4)	148(1)	Intramolecular
	C13–Br2···O1			2.941(3)	163.9(1)	x, y, z
**Compound-C**	N1–H1B···O1	0.82(4)	2.18(3)	3.002(5)	177(7)	2-x,-y,2-z
	N1–H1A···O2	0.92(6)	2.56(6)	3.381(6)	149(5)	1-x,-y,1-z
	O2–H2A···N2	0.76(6)	1.84(6)	2.561(5)	159(6)	Intramolecular
	C13–I2···O1			2.995(4)	161.9(1)	Intermolecular

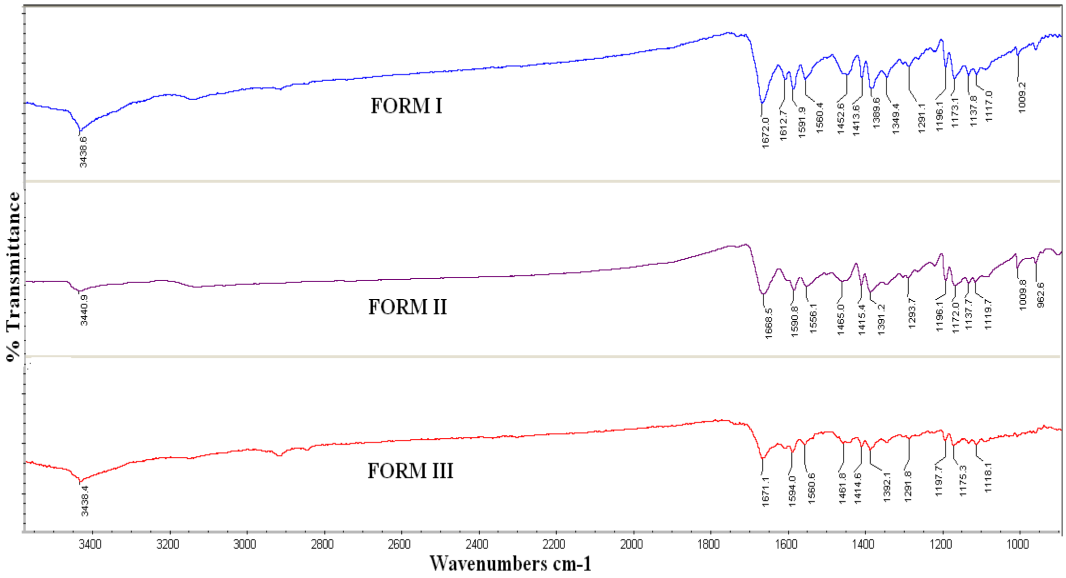

Figure S23 Comparison of IR stretching frequency for all the three polymorphs of compound-A.

Table S4 Stabilization Energy Values.

Compound	N-H…O (Å)	Stabilization Energy (K.cal/Mol)	C-X…O (Å)	Stabilization Energy (K.cal/Mol)
Compound-A (Form I)	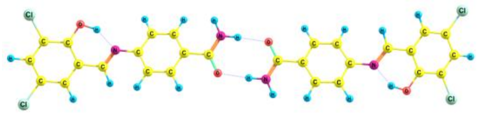 2.00	-15.20	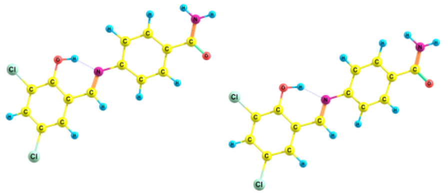 2.96	-0.07
Compound-B	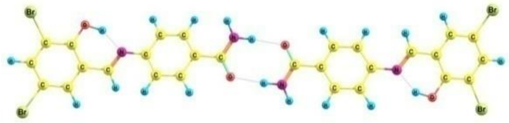 2.12	-14.75	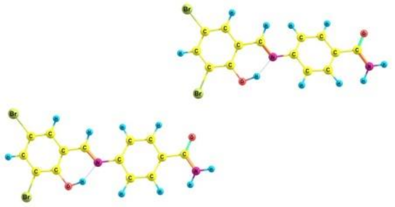 2.94	-0.52
Compound-C	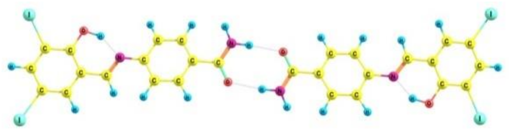 2.18	-14.77	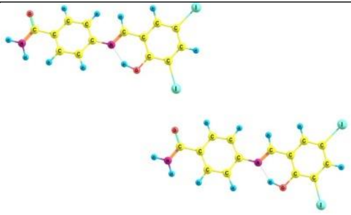 2.99	-1.7

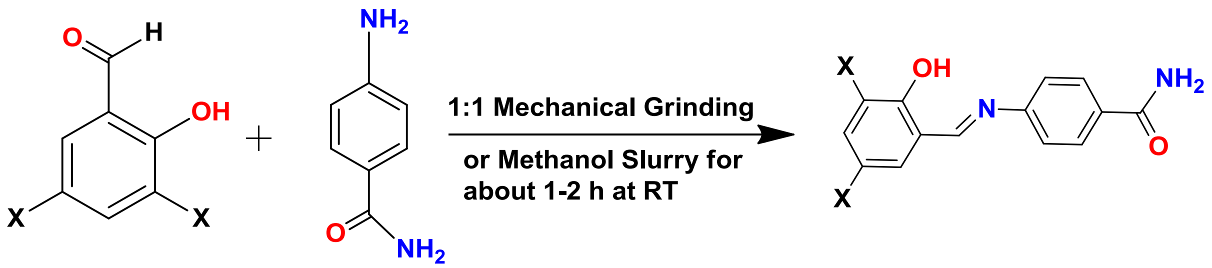

X = Cl, Br, I

Scheme S1 Synthesis of *N*-salicylideneanilines studied in the present work.

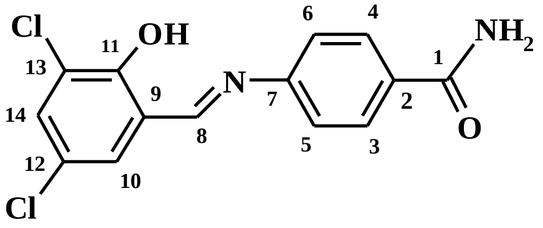

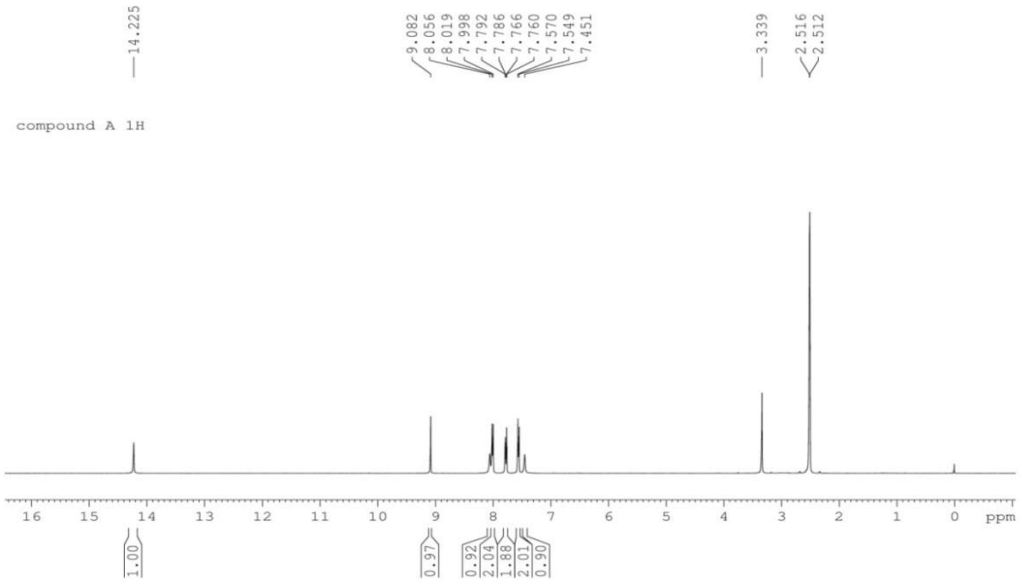

Figure S24 1H NMR spectrum of compound-A.

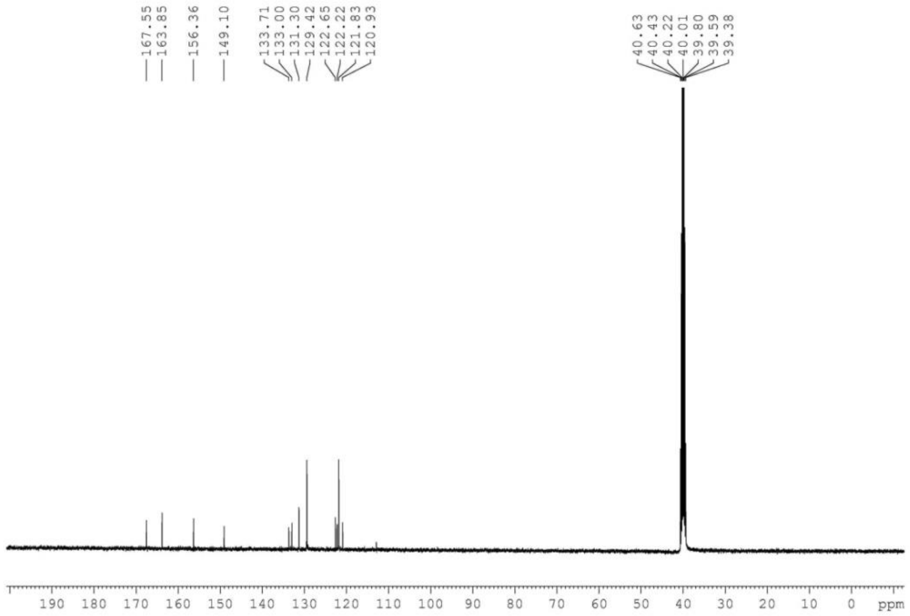

Figure S25 13C-NMR spectrum of compound-A.

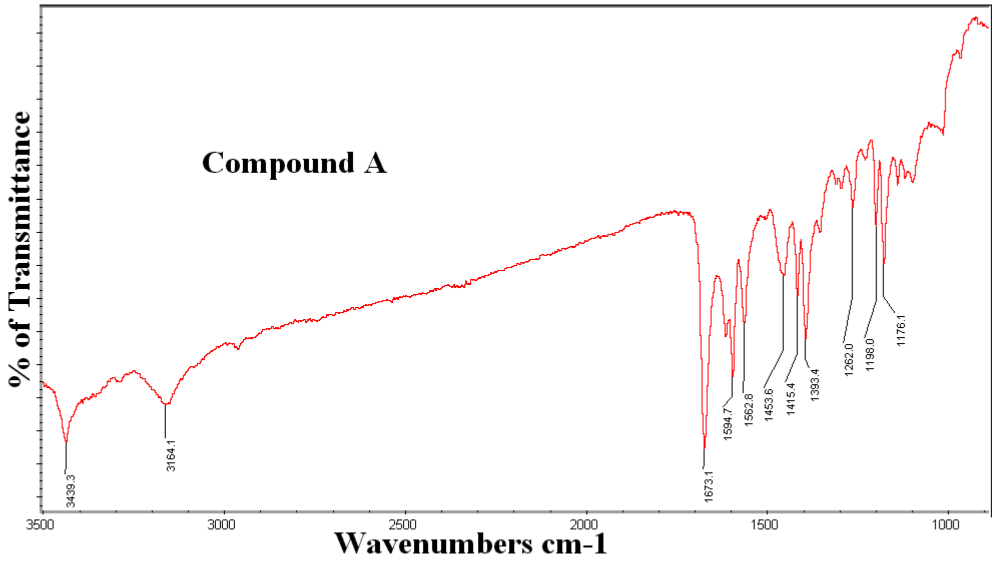

Figure S26 IR spectrum of compound-A.

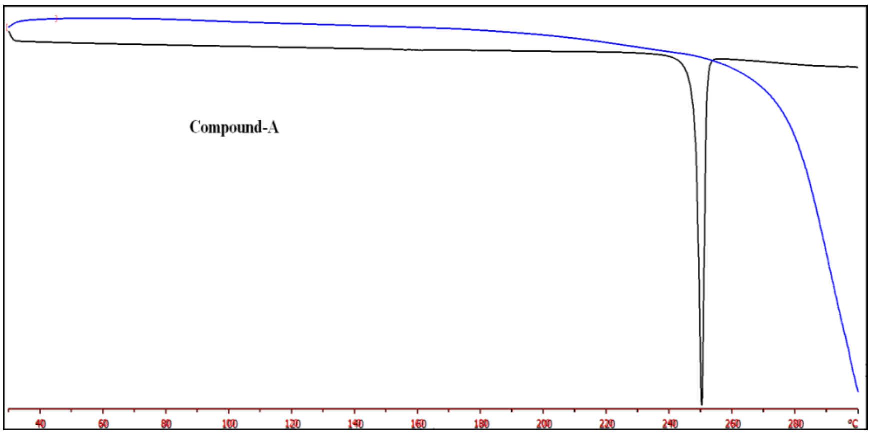

Figure S27 TGA and DSC of compound-A.

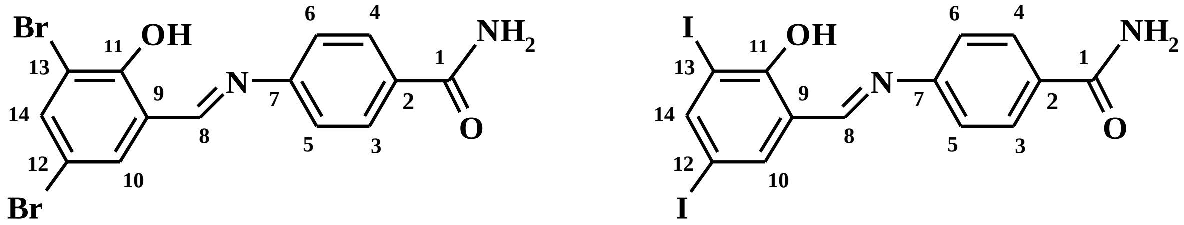

Compound-B Compound-C

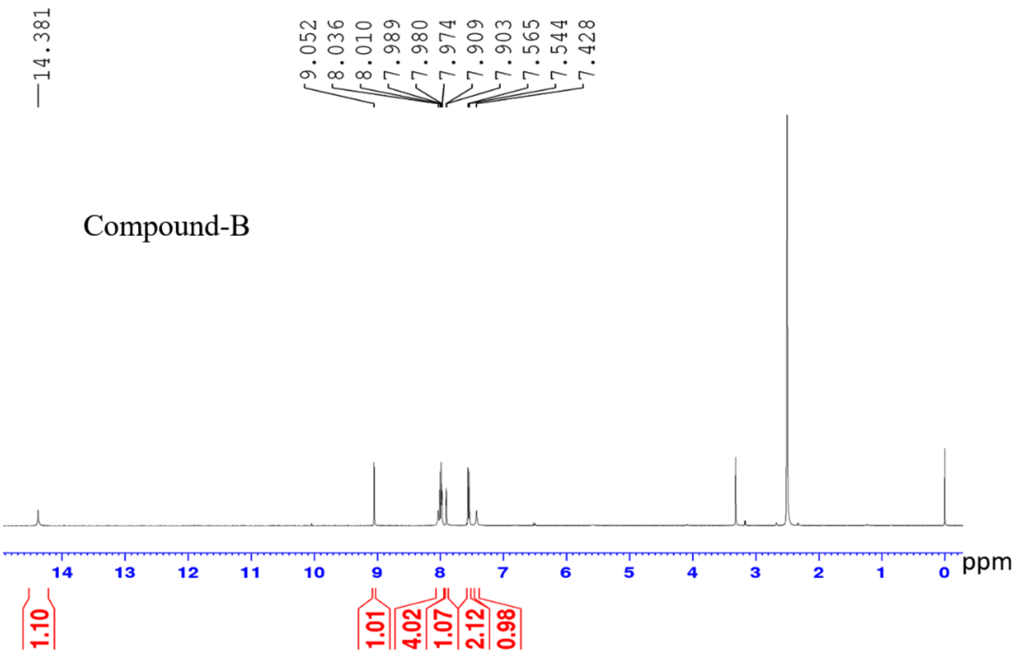

Figure S28 1H-NMR spectra for Compound-B.

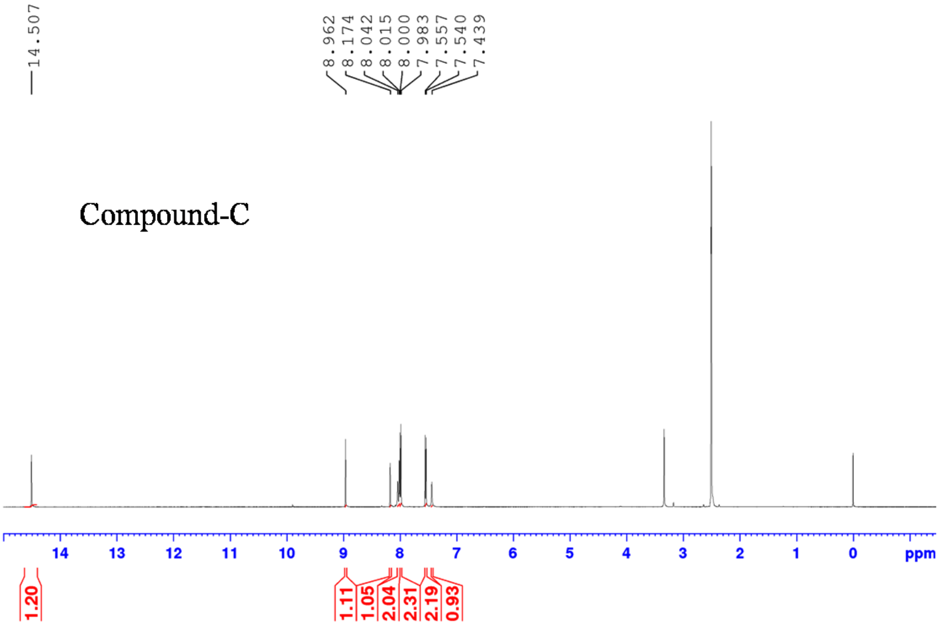

Figure S29 1H-NMR spectra for Compound-C.

References

Cohen, M. D., Schmidt, G. M. J. & Flavian, S. (1964). *J. Chem. Soc.* 2041–2051.

Hadjoudis, E. & Mavridis, I. M. (2004). *Chem. Soc. Rev*. **33**, 579–588.

Haneda, T., Kawano, M., Kojima, T. & Fujita, M. (2007). *Angew. Chem. Int. Ed*. **46**, 6643–6645.

Hutchins, K. M., Dutta, S., Loren, B. P. & MacGillivray, L. R. (2014). *Chem. Mater*. **26**, 3042–3044.

Ogawa, K., Kasahara, Y., Ohtani, Y. & Harada, J. (1998). *J. Am. Chem. Soc.* **120**, 7107–7108.

Senier, A. & Shepheard, F. G. (1909). *J. Chem. Soc. Trans.* **95**, 441–445.
